# Antibacterial and detoxifying activity of NZ17074 analogues with multi-layers of selective antimicrobial actions against *Escherichia coli* and *Salmonella enteritidis*

**DOI:** 10.1038/s41598-017-03664-2

**Published:** 2017-06-13

**Authors:** Na Yang, Xuehui Liu, Da Teng, Zhanzhan Li, Xiumin Wang, Ruoyu Mao, Xiao Wang, Ya Hao, Jianhua Wang

**Affiliations:** 1 Key Laboratory of Feed Biotechnology, Ministry of Agriculture, Beijing, 100081 China; 2 Gene Engineering Laboratory, Feed Research Institute, Chinese Academy of Agricultural Sciences, Beijing, 100081 China; 3 Institute of Biophysics, Chinese Academy of Sciences, Beijing, China

## Abstract

NZ17074 (N1), an arenicin-3 derivative isolated from the lugworm, has potent antibacterial activity and is cytotoxic. To reduce its cytotoxicity, seven N1 analogues with different structures were designed by changing their disulfide bonds, hydrophobicity, or charge. The “rocket” analogue-N2 and the “kite” analogue-N6 have potent activity and showed lower cytotoxicity in RAW264.7 cells than N1. The NMR spectra revealed that N1, N2, and N6 adopt β-sheet structures stabilized by one or two disulfide bonds. N2 and N6 permeabilized the outer/inner membranes of *E*. *coli*, but did not permeabilize the inner membranes of *S*. *enteritidis*. N2 and N6 induced *E*. *coli* and *S*. *enteritidis* cell cycle arrest in the I-phase and R-phase, respectively. In *E*. *coli* and in *S*. *enteritidis*, 18.7–43.8% of DNA/RNA/cell wall synthesis and 5.7–61.8% of DNA/RNA/protein synthesis were inhibited by the two peptides, respectively. Collapsed and filamentous *E*. *coli* cells and intact morphologies of *S*. *enteritidis* cells were observed after treatment with the two peptides. Body weight doses from 2.5–7.5 mg/kg of N2 and N6 enhanced the survival rate of peritonitis- and endotoxemia-induced mice; reduced the serum IL-6, IL-1β and TNF-α levels; and protected mice from lipopolysaccharide-induced lung injury. These data indicate that N2 and N6, through multiple selective actions, may be promising dual-function candidates as novel antimicrobial and anti-endotoxin peptides.

## Introduction

*Escherichia coli* and *Salmonella enteritidis* are major food-borne pathogens that often cause outbreaks of diarrheal diseases and extraintestinal infections in both animals and humans throughout the world^1^. The World Health Organization (WHO) has estimated that 1.7–2.5 million human deaths occur worldwide every year^2^. Antibiotics such as β-lactams, fluoroquinolones, aminoglycosides, and trimethoprim-sulfamethoxazole are the primary antimicrobials used to treat these infectious diseases^2–4^. However, resistance of *E*. *coli* and *S*. *enteritidis* to these antibiotics is definitively on the rise^3, 5^. Treatment with these antibiotic therapies also causes a three- to 20-fold increase in the total concentration of lipopolysaccharide (LPS) rapidly released from cell walls of gram-negative bacteria, and subsequently induces a variety of acute and chronic diseases including sepsis, fatal septic shock, ulcerative colitis, and systemic inflammatory response syndrome^6–8^. The annual incidence of sepsis has been roughly estimated at 15–19 million cases globally, and the mortality of severe sepsis reaches 30–50% worldwide^9, 10^. Although monoclonal antibodies or other antiendotoxin chemical drugs have been examined in a few clinical studies to combat LPS-induced diseases, these therapies have been largely unsuccessful due to either high host toxicity or limited effects^11, 12^. The absence of efficient therapies makes studies seeking novel LPS-neutralizing agents of the utmost importance.

The removal of gram-negative bacteria by antimicrobial peptides (AMPs) may be an effective strategy to control and prevent worsening antibiotic resistance and LPS-induced pathophysiological responses^8, 13^. It has been demonstrated that several α-helical AMPs, such as cathelicidin (LL-37) and KSL-W, can effectively neutralize *Porphyromonas gingivalis* and *E*. *coli* LPS and inhibit LPS-induced inflammatory cytokine production *in vitro* ^13, 14^. Bedran *et al*.^15^ reported that LL-37 and β-sheet peptide hBD-3 displayed anti-inflammatory activities when applied individually and synergistically to a three-dimensional co-culture model of gingival epithelial cells and fibroblasts^15^. Due to their small sizes (<30 amino acid residues) and excellent binding ability to the lipid bilayer of bacterial membranes, β-sheet AMPs such as s-thanatin are also well-established as promising candidates for the treatment of multidrug-resistant bacterial infections and neutralization of LPS^16, 17^.

Recent studies have mainly focused on developing β-sheet AMPs that have fewer or no Cys residues, as they are easier and less expensive to produce^18^. It has been reported that there are three main categories of β-sheet AMPs that have: (i) antimicrobial activity that is independent of the number of disulfide bonds, for example human β-defensins 1–3, cryptdin-4, μ-conotoxins, and thanatin^19–23^; (ii) have enhanced antimicrobial activity in the presence of fewer disulfide bonds such as HNP-1 and cryptdin-4 variants^19, 24^; and (iii) have decreased antimicrobial activity in the presence of fewer or no disulfide bonds, such as PM1-S, human β-defensin 3, human α-defensin HNP-1, and arenicin-1^25, 26^. This difference is most likely related to the position of cationic residues in the disulfide loop of AMPs. For example, the location of “K” or “R” residues in disulfide bonds could be an important determining factor for their antimicrobial activity^20^. On the other hand, intramolecular disulfide bond locations of several AMPs are also very important for antimicrobial activity^27^. Harwig *et al*. designed PG-1 variants that lacked one pair of disulfide bonds - a “bullet” analogue (Cys → Ala at position of 8 and 13, disulfide bond distal to the turn) - and a “kite” analogue (Cys → Ala at position 6 and 15, disulfide bond proximal to the turn) and found that, compared with PG-1, the “kite” analogue had a greater loss of antimicrobial activity than the “bullet” analogue^27^.

Marine peptide NZ17074 (N1), a variant of arenicin-3 (Tyr5 → Asn, Tyr17 → His) isolated from the marine invertebrate lugworm *Arenicola marina*, exhibits potent antimicrobial activity against gram-negative bacteria and fungi, and it is currently undergoing preclinical studies^28–30^. However, N1 has a significant degree of toxicity in normal eukaryotic cells^29, 30^. Additionally, the role of disulfide bonds in modulating the antimicrobial activity of N1 is unclear. Thus, modification of N1 is required to generate more therapeutically valuable molecules. It has been found that several residues (Val6, 8, 13, 15, Tyr9, and Asn11, 21) of N1 are essential for antibacterial activity^31^. In the present study, to reduce cell cytotoxicity, determine the role of disulfide bonds, evaluate structure–activity relationships and improve antimicrobial activity, modification of N1 was performed based on the replacement of multiple residues by the hydrophobic/cationic residues Ala/Lys and the removal of disulfide bonds or the addition of hydrophobic Trp residue at the 1st, 3rd, 4th, 5th, 7th, 12th, 16th, and 20th positions, which generated four types of N1 variants: (i) “rocket” analogues with two disulfide bonds (Gly1,12 → Ala; Trp4, Asn5 → Ala), (ii) a “kite” analogue with one disulfide bond (Cys3,20 → Ala), (iii) a “bullet” analogue with one disulfide bond (Cys7,16 → Ala), and iv) other linear analogues without disulfide bonds (∆Cys3,7,16,20; Cys3,7,16,20 → Ala). These peptides were chemically synthesized and their antimicrobial activity against gram-negative bacteria was examined. Multiple possible levels of selective antimicrobial actions of N2 and N6 against *E*. *coli* and *S*. *enteritidis* were further elucidated. Additionally, the *in vivo* antibacterial activity of N2 and N6 was evaluated in murine peritonitis models induced by *E*. *coli* and *S*. *enteritidis*, respectively, as well as endotoxin neutralizing activity in a murine endotoxemia model challenged with LPS.

## Results

### Peptides designed based on structural determinants

Seven N1 analogues were obtained by changing disulfide bonds, hydrophobicity, or charges. According to the number and location of disulfide bonds, they are divided into the following classes: “rocket” analogues with two disulfide bonds (N2: Gly1,12 → Ala; N3: Trp4,Asn5 → Ala; N4: Trp0), a “kite” analogue with a Cys7-Cys16 disulfide bond (N6: Cys3,20 → Ala), a “bullet” analogue with a Cys3-Cys20 disulfide bond (N7: Cys7,16 → Ala), and other disulfide bond-free linear analogues (N5: ∆Cys3,7,16,20; N8: Cys3,7,16,20 → Ala) (Supplementary Fig. S1). As shown in Table 1, the PI, charge, hydrophilicity, aliphatic index, antibacterial activity score, and α-helix values of N4 were highly similar to those of the parent peptide-N1. The net charge of N3 was higher than that of N1 and the other analogues. The N3, N6, N7, and N8 peptides had a higher α-helix content (28.6%), and N2 and N5 contained a lower α-helix content (9.5% and 11.8%) than N1. The β-sheet contents of N5 to N8 deceased from 71.4% to 57.1%. Hydrophobicity of N2 and N3 increased from −0.133 to −0.033, indicating that they are more hydrophobic than N1 due to the addition of a methyl group by Gly/Gln to Ala residue substitution at positions 1, 5, and 12. The aliphatic indices of N2 to N8, with the exception of N4, ranged from 60.00 to 74.29, which were higher than that of N1. The Boman index of the N1 analogues, except for N5, deceased from 2.61 to 2.43 kcal/mol, indicating higher antibacterial activity. N3 had the highest antibacterial activity score, while N5 and N8 had the lowest score.

**Table 1:** Sequences and physicochemical properties of N1 and its analogues.

	Amino acid sequence	MW (Da)	PI	Charge	GRAVY	AI	BI (kcal/mol)	AAS	Secondary structure (%)	
									α-helix	β-sheet
Parent peptide										
N1 (NZ17074)	GFCWNVCVYRNGVRVCHRRCN	2452.0	9.37	+4	−0.243	55.24	2.66	0.245	0	81.0
Hydrophobicity/charge modification: “rocket”										
N2	AFCWNVCVYRNAVRVCHRRCN	2570.0	9.38	+4	−0.033	64.76	2.58	0.121	9.5	81.0
N3	GFCKAVCVYRNGVRVCHRRCN	2440.9	9.69	+5	−0.133	60.00	2.63	0.413	28.6	81.0
N4	WGFCWNVCVYRNGVRVCHRRCN	2728.1	9.37	+4	−0.273	52.73	2.43	0.273	0	86.4
S-S modification: “kite”/“bullet”/“linear”										
N5	GF_WNV_VYRNGVRV_HRR_N	2129.4	12.00	+4	−0.888	68.24	3.59	−0.053	11.8	70.6
N6	GFAWNVCVYRNGVRVCHRRAN	2477.8	10.72	+4	−0.310	64.76	2.61	0.229	28.6	71.4
N7	GFCWNVAVYRNGVRVAHRRCN	2477.8	10.72	+4	−0.310	64.76	2.61	0.229	28.6	66.7
N8	GFAWNVAVYRNGVRVAHRRAN	2413.7	12.00	+4	−0.376	74.29	2.56	−0.007	28.6	57.1

Note: MW: molecular weight; PI: isoelectric point; GRAVY: grand average of hydropathicity; underlined residues: the modified residues. AI: aliphatic index; BI: Boman index; AAS: antibacterial activity score; S-S: disulfide bonds. N1, N2, N3 and N4: the “rocket” analogues with two disulfide bonds of Cys3-20 and Cys7-16; N6: the “kite” analogue with one disulfide bond of Cys7-16; N7: the “bullet” analogue with one disulfide bond of Cys3-20; N5 and N8: the other analogues without disulfide bonds.

### N2/N6 have potent antimicrobial activity, low hemolysis and cytotoxicity, and no antimicrobial resistance

*Antimicrobial activity*. As shown in Table 2, the “rocket” N2 analogue, with two disulfide bonds (Gly1,12 → Ala), exhibited the strongest antimicrobial activity against gram-negative bacteria with minimal inhibitory concentration (MIC) values ranging from 0.25 to 1 μg/ml against *E*. *coli*, *Salmonella*, and *Pseudomonas* strains. Compared to N1, the “rocket” N3 (Trp4,Asn5 → Ala) showed relatively weaker antimicrobial activity against bacteria and *Candida albicans*. The additional introduction of Trp1 did not significantly affect antimicrobial activity of the “rocket” N4, indicating that Trp1 is not important for antimicrobial activity. Disulfide-free linear N5 (∆Cys3,7,16,20) had the lowest antimicrobial activity against *Salmonella*, *Pseudomonas*, and *S*. *suis* (with MICs > 64 μg/ml), which may be related to its higher hydrophilicity. The antimicrobial activity of the “kite” N6 (Cys3,20 → Ala) with one disulfide bond, was similar to that of N1; the “bullet” N7 (Cys7,16 → Ala) with one disulfide bond, had higher antimicrobial activity against several gram-positive bacteria than N1, but lower activity against gram-negative bacteria. Alanine substitution of four Cys residues largely decreased the antimicrobial activity of N8 (Cys3,7,16,20 → Ala) against bacteria, with an approximate 4–128-fold increase in MICs. This indicated that a single disulfide bond was sufficient to restore antimicrobial potency and that the Cys7-Cys16 disulfide bond rather than the Cys3-Cys20 one is indispensable for N1 antimicrobial activity.

**Table 2:** The MIC values of N1 and its analogues.

Strains	MIC (μg/ml)							
	N1	N2	N3	N4	N5	N6	N7	N8
Gram-negative bacteria								
*Escherichia coli* CVCC195^a^	0.5–1	0.25	1	1	64	0.5	2	32
*E*. *coli* CVCC1515^a^	0.25	0.5	1	1	128	1	2	16
*E*. *coli* CICC21530 (serotype O157:H7)^b^	0.5	0.25	1	1	64	0.5	2	16
*Salmonella typhimurium* ATCC14028^c^	0.5	1	4	2	128	2	8	>64
*S*. *enteritidis* CVCC3377^a^	0.25	0.125	0.5	0.25	4	0.25	0.5	1
*S*. *pullorum* CVCC1789^a^	0.25	0.5	2	1	>128	0.5	4	>128
*S*. *pullorum* CVCC1802^a^	0.25	0.25	1	2	>128	0.5	4	32
*S*. *choleraesuis* CVCC503^a^	0.5	0.5	4	NA	>128	2	16	>64
*Pseudomonas aeruginosa* CICC10419^b^	2	2	>16	NA	>128	8	>64	>128
*P*. *aeruginosa* CICC21630^b^	4	2	16	4	>128	4	>64	>128
Gram-positive bacteria								
*Staphylococcus aureus* ATCC43300^c^	16	16	>16	8	>128	16	>64	>64
*S*. *aureus* ATCC25923^c^	0.5–1	0.25	0.25	1	2	0.25	0.5	2
*S*. *suis* CVCC606^a^	16	16	16	NA	>128	16	8	>128
*Enterococcus faecium* CMCC1.2136^d^	>16	4	8	NA	64	4	2	16
*Bacillus subtilis* ATCC6633^c^	0.25	0.5	2	1	64	0.5	2	4
*Listeria ivanovii* ATCC19119^c^	128	4	8	NA	128	NA	2	NA
Fungi								
*Candida albicans* CGMCC2.2411^d^	16	32	>16	NA	>128	64	>16	NA

Note: NA: no detection. ^a^China Veterinary Culture Collection Center (CVCC); ^b^China Center of Industrial Culture Collection (CICC); ^c^American Type Culture Collection (ATCC); ^d^National Center Center for Medical Culture Collection (CMCC). Data were representative of three independent experiments.

#### Low hemolysis, cytotoxicity, and no antimicrobial resistance

**Figure 1:**
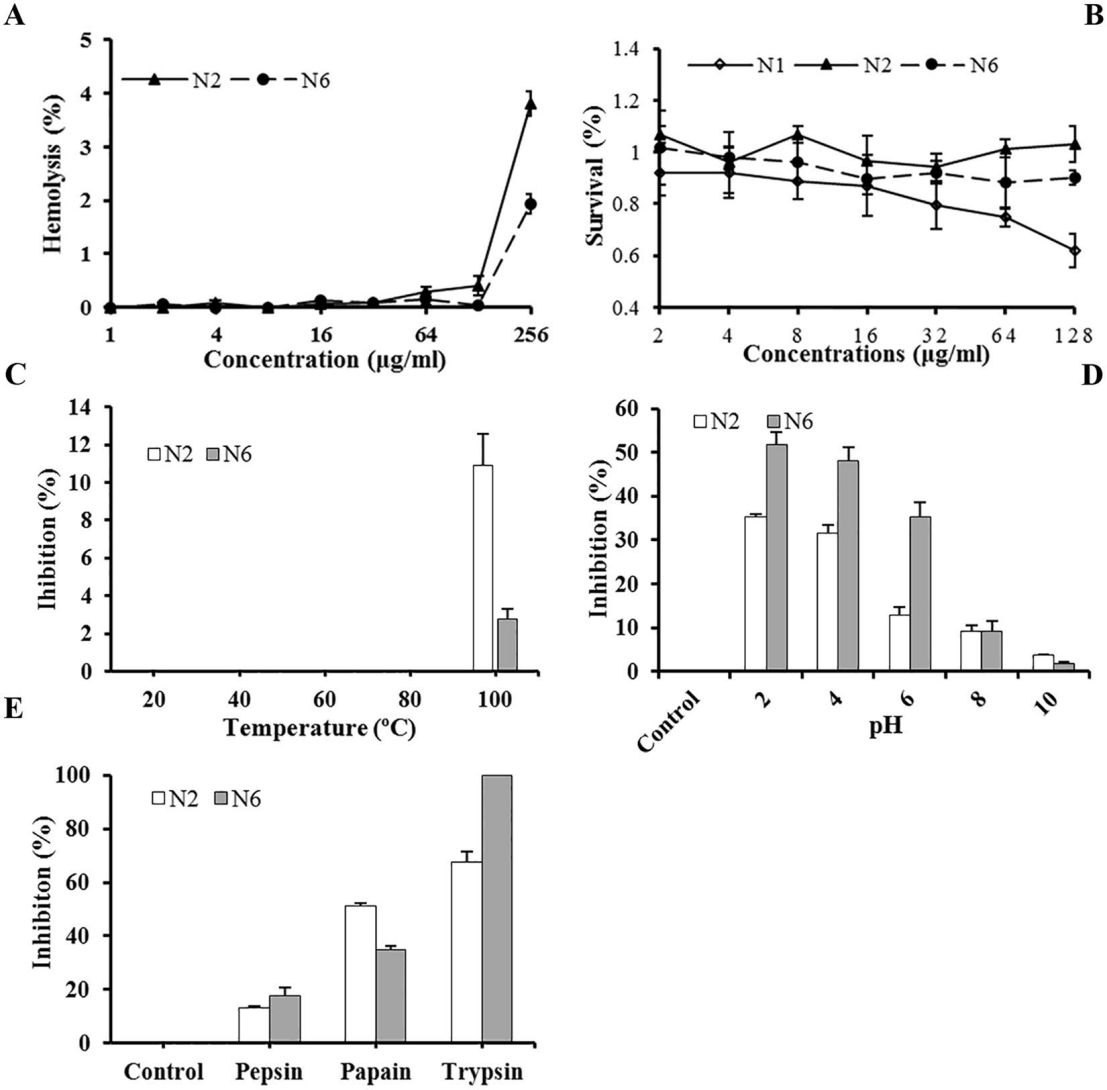
Hemolytic activity, cytotoxicity and stability of N2 and N6. (**A**) Hemolytic activity of N2 and N6 against murine erythrocytes. (**B**) Cytotoxicity of the peptides against RAW264.7 cells. (**C**,**D**,**E**) The effects of temperature (**C**), pH (**D**) and proteases (**E**) on the antibacterial activity of N2 and N6 *in vitro* against *S*. *enteritidis* CVCC3377 cells. The results are given as the mean ± SEM (n = 3).

As shown in Fig. 1A, the hemolysis of N2 and N6 at a concentration of 128 μg/ml was 0.4% and 0.04%, respectively, which was lower than that of the parent peptide N1 (3%)^29^. Even at a concentration of 256 μg/ml, the hemolysis of N2 and N6 was 3.8% and 1.9%, respectively, indicating that the two peptides have very low hemolytic activity against murine erythrocytes. The cytotoxicity of N2 and N6 in murine peritoneal RAW264.7 macrophage cells was determined via the 3-(4,5-dimethylthiazol-2-yl)-2,5-diphenyl tetrazolium bromide (MTT) assay (Fig. 1B). The cell survival of N2 and N6 at a concentration range of 64–128 μg/ml was higher than that of N1, indicating that both N2 and N6 have lower cytotoxicity than N1 (from 25.3% to 37.9%). N2 demonstrated the lowest cytotoxicity (from 3.3% to 5.8%) and N6 (from 9.6% to 11.8%) had moderate cytotoxicity. These results suggested that the application of N2 and N6 at high concentrations can cause mild disruption of eukaryotic cells.

Additionally, after 18 serial passages in the presence of N2 or N6, the MIC values did not change and no mutants of *E*. *coli* resistant to N2 or N6 were observed (data not shown).

### N2/N6 exhibited adequate thermal, alkaline pH, and anti-pepsin stability

The antimicrobial activity of N2 and N6 against *E*. *coli* and *S*. *enteritidis* was not affected upon exposure to 20, 40, 60, or 80 °C temperatures, but a small reduction of 2.2–16.5% for *E*. *coli* and of 2.8–10.9% for *S*. *enteritidis* was observed after incubation at 100 °C for 1 h (Fig. 1C, Supplementary Fig. S2A). N2 and N6 were stable between pH 8.0 and pH 10.0, but very susceptible to disruption between pH 2.0 and pH 4.0 (Fig. 1C, Supplementary Fig. S2B). Additionally, the inhibitory activity of N2 and N6 decreased by 40–100% for *E*. *coli* and by 51.4–100% for *S*. *enteritidis* after incubation for 4 h with trypsin and papain, and by 20.3–26.4% for *E*. *coli* and 13.3–17.7% for *S*. *enteritidis* after treatment with pepsin (Fig. 1E, Supplementary Fig. S2C).

### Structures of N2/N6 analyzed by circular dichroism (CD) and nuclear magnetic resonance (NMR)

#### CD

**Figure 2:**
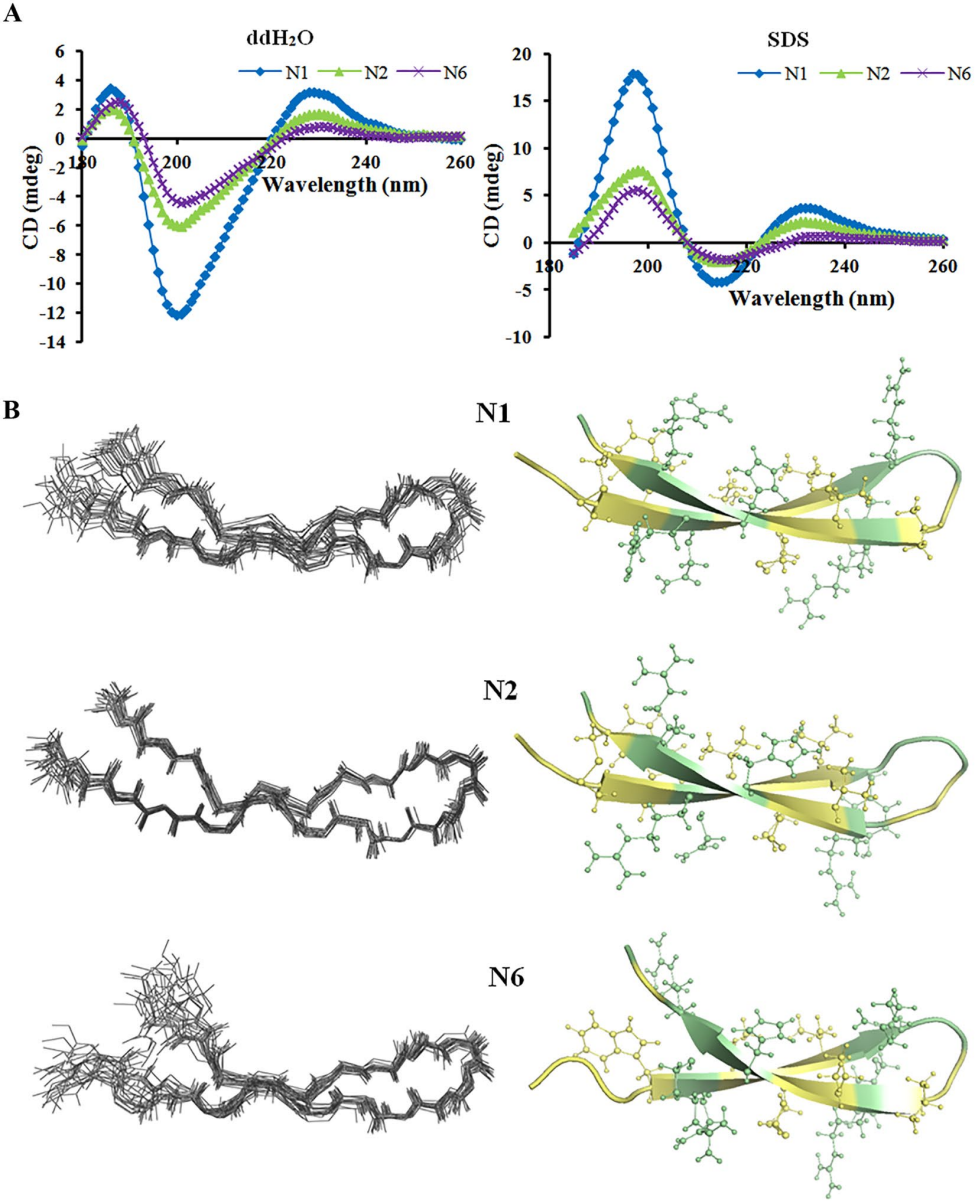
Structures of N1, N2, and N6. (**A**) CD spectra of N1, N2 and N6. (**B**) NMR analysis of N1, N2 and N6. Left panel shows the sets of the 20-best structures, superimposed over the backbone atoms. Only backbone atoms are shown. Right panel shows the ribbon representations of the peptides. The side chains on the β-sheet strands are represented by balls and sticks. The hydrophobicity and hydrophilicity of the residues are color-coded pale yellow and pale-green, respectively. The figure was prepared using the program Pymol.

The anionic surfactant sodium dodecyl sulfate (SDS) is often used to mimic the cell membrane environment. To analyze the structural features of N2/N6, the CD spectra of peptides were measured in ddH_2_O and 10–40 mM SDS, respectively. The secondary structures of N2/N6 in ddH_2_O were characterized predominantly by antiparallel strands (56.4–57.9%), α-helices (19.6–22.0%), and β-turns (21.2–21.9%) with a characteristic positive maximum at 190 nm and 230 nm and negative minimum at 205 nm (Fig. 2A). Both N2 and N6 showed a significant increase in α-helices (>80.3%), higher than the parent peptide N1 (<54.6%), and decrease in antiparallel strands (<0.8%) in SDS (Supplementary Table S1). This result indicated that SDS is favorable to form α-helical structures.

#### NMR

From 100 calculated structures, 20 with the lowest energy were selected to undergo water refinement, and the refined structures were used to represent the peptide structure in aqueous solution. The NMR-determined structures of N1, N2 and N6 are shown in Fig. 2B as superimposed backbone traces of the ensembles and their representative ribbon diagrams. The ribbons were generated using model 2 of N1, model 20 of N2 and model 19 of N6, which were the medoid structures of each ensemble. The structural statistics are listed in Table 3. Not surprisingly, N1, N2 and N6 adopt a β-hairpin structure (Fig. 2B) stabilized by one (N6, Cys7-Cys16) or two (N1 and N2, Cys7-Cys16 proximal to the β-sheet, and Cys3-Cys20 distal to the β-sheet) disulfide bonds and several inter-strand hydrogen bonds, which are determined both in the structure calculation process and by analyzing the temperature coefficients (Supplementary Table S2). The antiparallel β-strands are Cys3-Arg10 and Val13-Cys20 for N1, Cys3-Try9 and Arg14-Cys20 for N2, and Trp4-Arg10 and Val13-Arg19 for N6 (Fig. 2B). The Gly to Ala mutation in N2 disturbs the tip of the β-hairpin and twists the connecting loop slightly, possibly to shield the larger hydrophobic side chain of Ala. Supplementary Fig. S3 illustrates the surface of each peptide with composing amino acids color-coded as hydrophobic or hydrophilic. N1, N2 and N6 showed impressive characteristics of amphipathicity. The hydrophobic side is dominated by F2, W4, V6, C7 and V8, while the hydrophilic side is composed mainly of R10, N11, H17, R18, R19 and N21. The electrostatic map indicated that N1 and N6 possess large cationic areas with very limited negative charge (Supplementary Fig. S4). However, N2 somehow has a greater negative charge and a disrupted positive-charge area. The absence of one disulfide bond in N6 shortens each of the β-sheet-forming strands by one residue (A3 and A20) and makes the residues at the N and C terminal more flexible, as suggested by larger amide proton temperature coefficients (Supplementary Table S2). The double disulfide bonds do not guarantee a more thermo-stable structure, as seen in Fig. 1C. However, these analogues do show pH stability in an alkaline pH range (Fig. 1D) and more protection against pepsin and papain but not trypsin.

**Table 3:** Structure statistics for 20 conformers of the three peptides N1, N2 and N6.

	N1	N2	N6
Unambiguous distance restraints			
Intrar-residue(i-j = 0)	150	169	115
Sequential (|i-j| = 1)	68	85	81
Medium range (2 ≤ |i-j| ≤ 4)	16	37	19
Long range (|i-j| ≥ 5)	51	75	34
Ambiguous distance restraints	6	11	10
Hydrogen bond	9	7	3
Disulfide bond	2	2	1
Total	302	386	263
Dihedral angle restraints			
Φ	17	17	17
Ψ	17	17	17
Total	34	34	34
Mean r.m.s. deviations from the experimental restraints			
Distance (Å)	1.73e-2 ± 5.48e-3	2.27e-2 ± 2.65e-3	2.54e-2 ± 3.25e-3
Dihedral angle (°)	2.14e-1 ± 1.14e-1	3.23e-1 ± 8.70e-2	4.30e-1 ± 1.09e-1
Mean r.m.s. deviations from idealized covalent geometry			
Bond (Å)	3.57e-3 ± 2.24e-4	3.99e-3 ± 2.03e-4	3.97e-3 ± 1.99e-4
Angle (°)	4.01e-1 ± 2.28e-2	4.78e-1 ± 2.12e-2	4.41e-1 ± 2.64e-2
Improper (°)	8.82e-1 ± 1.43e-1	1.03 ± 1.23e-1	1.04 ± 1.37e-1
Ramachandran plot			
most favorable regions	92.6%	87.9%	85.9%
additional allowed regions	7.4%	12.1%	14.1%
generously allowed regions	0%	0%	0%
Atomic r.m.s. differences (Å)^b^			
Backbone heavy atoms	0.76 (0.54)	0.49 (0.19)	1.29 (0.20)
All heavy atoms	1.27 (1.15)	0.93 (0.75)	1.94 (0.95)

None of the structures exhibits distance violations greater than 0.3 Å or dihedral angle violations greater than 4˚. ^a^The program PROCHECK (http://www.biochem.ucl.ac.uk/~roman/procheck_nmr/procheck_nmr.html) was used to assess the overall quality of the structures; ^b^The numbers in the parenthesis are the rms differences for the well-ordered region (residues 3–20 for N1, residues 2–20 for N2, and residues 3–19 for N6).

### N2/N6 permeabilized model membranes and cell membranes and disrupted membrane potentials

#### Model membranes

**Figure 3:**
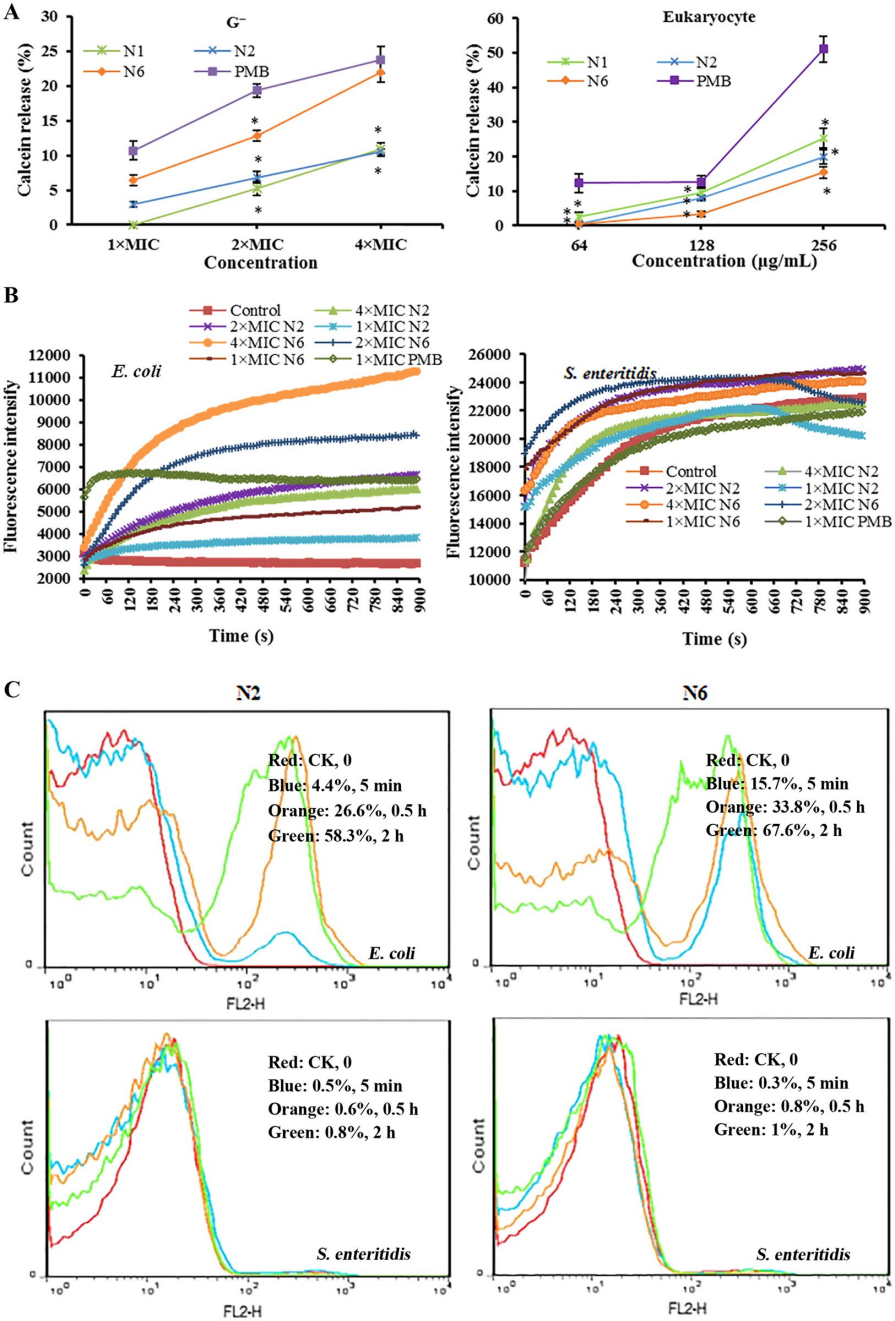
Penetration of biofilms by N2 and N6. (**A**) Calcein leakage from POPC/POPG = 4:1 (gram-negative) and POPC/cholesterol = 1:1 (eukaryotic) model membranes induced by different concentrations of N2 and N6, respectively. The results are given as the mean ± SEM (n = 3). Differences between groups were determined by one-way ANOVA followed by SPSS analysis. *p < 0.05 compared to the PMB group. (**B**) Outer membrane permeabilization assays of N2 and N6. (**C**) Flow cytometric analysis of PI-staining in *E*. *coli* CVCC195 and *S*. *enteritidis* CVCC3377 cells treated with 1× MIC N2 and N6, respectively. Red line: no peptide, negative control; Blue line: treatment with peptides for 5 min; Orange line: treatment with peptides for 0.5 h; Green line: treatment with peptides for 2 h.

To gain insights into the mechanism of action of N2 and N6, the release of the fluorescent dye calcein from model bacterial and mammalian membranes was measured with a Tecan Infinite M200 PRO microplate reader. As shown in Fig. 3A, N2 and N6 induced 3.0–10.5% and 6.3–21.9% dye leakage from phosphatidylcholine (POPC)/phosphatidylglycerol (POPG) large unilamellar vesicles (LUVs) (imitating bacterial cell membranes) at 1×, 2×, and 4× MIC against *E*. *coli* cells, which was higher than the parent peptide N1. N2 and N6 induced less dye leakage (0.6–19.9% and 0.4–15.4%) from POPC/cholesterol LUVs mimicking eukaryotic cell membranes at concentrations from 64 to 256 μg/ml compared to N1 (2.5–25.3%) and polymyxin B (PMB) (12.3–51.0%). This suggested that N2 and N6 have more potent abilities to permeabilize cell membranes of gram-negative bacteria and lower cytotoxicity against eukaryotic cells than N1.

#### Outer membrane permeability

The ability of peptides to permeabilize outer membranes was measured with the N-phenyl-1-naphthylamine (NPN) fluorescent dye. As shown in Fig. 3B, both N2 and N6 induced a time-dependent and concentration-dependent increase in NPN fluorescence in both *E*. *coli* and *S*. *enteritidis* cells. The addition of 1× and 2× MIC of N2 or N6 to *E*. *coli* cells caused a small increase in fluorescence intensity, indicating slow outer membrane permeability of peptides. However, permeabilization of *S*. *enteritidis* began within 1 min after treatment with N2 or N6 and a one-fold increase in fluorescence was observed, indicating that the two peptides can instantly permeabilize the outer membrane of *S*. *enteritidis* cells and that N6 has higher penetrating ability than N2.

#### Inner membrane permeability

The red fluorescent dye propidium iodide (PI), which is blocked on the outside of intact membranes, can penetrate damaged cell membranes and intercalate into nucleic acid. The fluorescent intensity of PI indicates the level of cell membrane integrity. In the absence of peptides, 99% of cells exhibited no PI staining, indicating that membranes were intact (Fig. 3C). After treatment with N2 and N6 for 5 min−2 h, the percentages of PI-permeable *E*. *coli* cells were 4.4–58.3% and 15.7–67.6%, respectively. This suggested that the inner membrane of *E*. *coli* is disrupted after 5 min of treatment with N2 and N6. However, both N2 and N6 did not disrupt the inner membrane of *S*. *enteritidis*, which showed <1% PI-staining.

#### Membrane potential

The changes in membrane potential were examined using rhodamine-123, which accumulates on the inner surface of intact membranes. If the membrane is disrupted, the potential dissipates and fluorescence diminishes^32^. As shown in Supplementary Fig. S5, cellular fluorescence of *E*. *coli* cells treated with N2 or N6 for 2 h was elevated in a concentration-dependent manner compared to that of control cells without treatment (CK). *S*. *enteritidis* cells treated with 1×, 2×, and 4× MIC N2 or N6 had a slight increase in cellular fluorescence. This result indicated that both N2 and N6 caused plasma membrane potential depolarization in *E*. *coli*, but that the inner membrane of *S*. *enteritidis* is intact and is not disrupted by N2 and N6, consistent with investigations of the inner membrane permeability.

#### Intracellular ROS accumulation

Reactive oxygen species (ROS) are a trigger that induce cell apoptosis and have destructive actions on DNA and proteins^30^. To determine if ROS are a mechanism of bacteria-killing, we used the ROS-sensitive dye dihydrorhodamine-123 (DHR-123) to monitor the production and accumulation of intracellular ROS after incubation with peptides. The results showed that N2 and N6 did not induce changes in fluorescence in *E*. *coli* (Supplementary Fig. S6A and B). However, *S*. *enteritidis* cells exhibited higher ROS levels than untreated cells after treatment with N2 or N6 (Supplementary Fig. S6C and D). This result indicated that N2 and N6 promoted ROS generation in *S*. *enteritidis* cells, which may be the main mechanism of killing them.

### Morphological changes observed by electron microscopy after treatment with N2/N6

#### Scanning electron microscopy (SEM) observations

**Figure 4:**
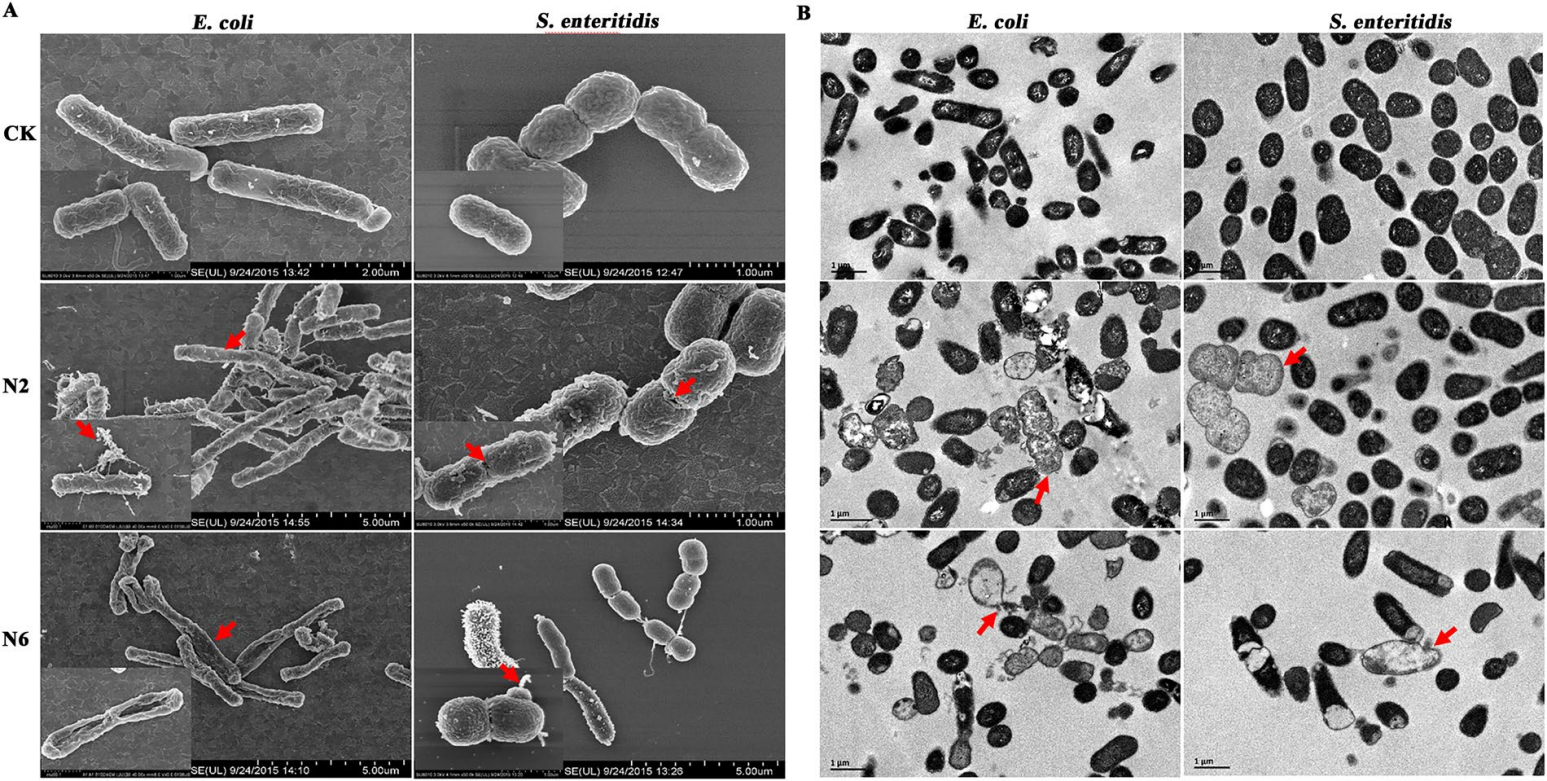
Electron microscopy analysis of *E*. *coli* CVCC195 and *S*. *enteritidis* CVCC3377 cells treated with N2 and N6. Bacteria in mid-logarithmic growth phases were treated with peptides at 4× MIC for 2 h. (**A**) SEM images of *E*. *coli* CVCC195 and *S*. *enteritidis* CVCC3377 cells treated with N2 and N6. (**B**) TEM images of *E*. *coli* CVCC195 and *S*. *enteritidis* CVCC3377 cells treated with N2 and N6.

*E*. *coli* CVCC195 and *S*. *enteritidis* CVCC3377 cells were treated for 2 h with 4× MIC N2 and N6, respectively, and observed by SEM to investigate cellular morphological changes. As shown in Fig. 4A, the non-treated *E*. *coli* cells exhibited intact smooth surfaces, while the cells treated with peptides underwent considerable structural changes. After treatment with N2, over 50% filamentous cells with filiferous substances outside of *E*. *coli* cells were produced. Collapsed and filamentous *E*. *coli* cells were observed after treatment with N6. For *S*. *enteritidis*, several holes on the outer membrane of cells were observed after treatment with N2 and N6, but overall cell morphology had no change.

#### Transmission electron microscopy (TEM) observations

The effects of N2 and N6 on *E*. *coli* CVCC195 and *S*. *enteritidis* CVCC3377 cells were also visualized using TEM. As shown in Fig. 4B, the untreated control *E*. *coli* CVCC195 and *S*. *enteritidis* CVCC3377 cells displayed normal morphology and intact cell membranes, and the cytoplasm appeared to have homogeneous electron density. After treatment with 4× MIC N2 or N6 for 2 h, cell membrane disruption and leakage of cellular contents (approximately 40%) were observed in *E*. *coli*. For *S*. *enteritidis*, cell swelling and ghost cells were observed after treatment with N2 and N6. The cytoplasm displayed a heterogeneous electron density, the cell morphology was deformed, and approximately 15% cell lysis occurred.

### N2/N6 bound to bacterial genomic DNA, disrupted cell cycles, and inhibited macromolecular synthesis

#### DNA gel movement retardation

**Figure 5:**
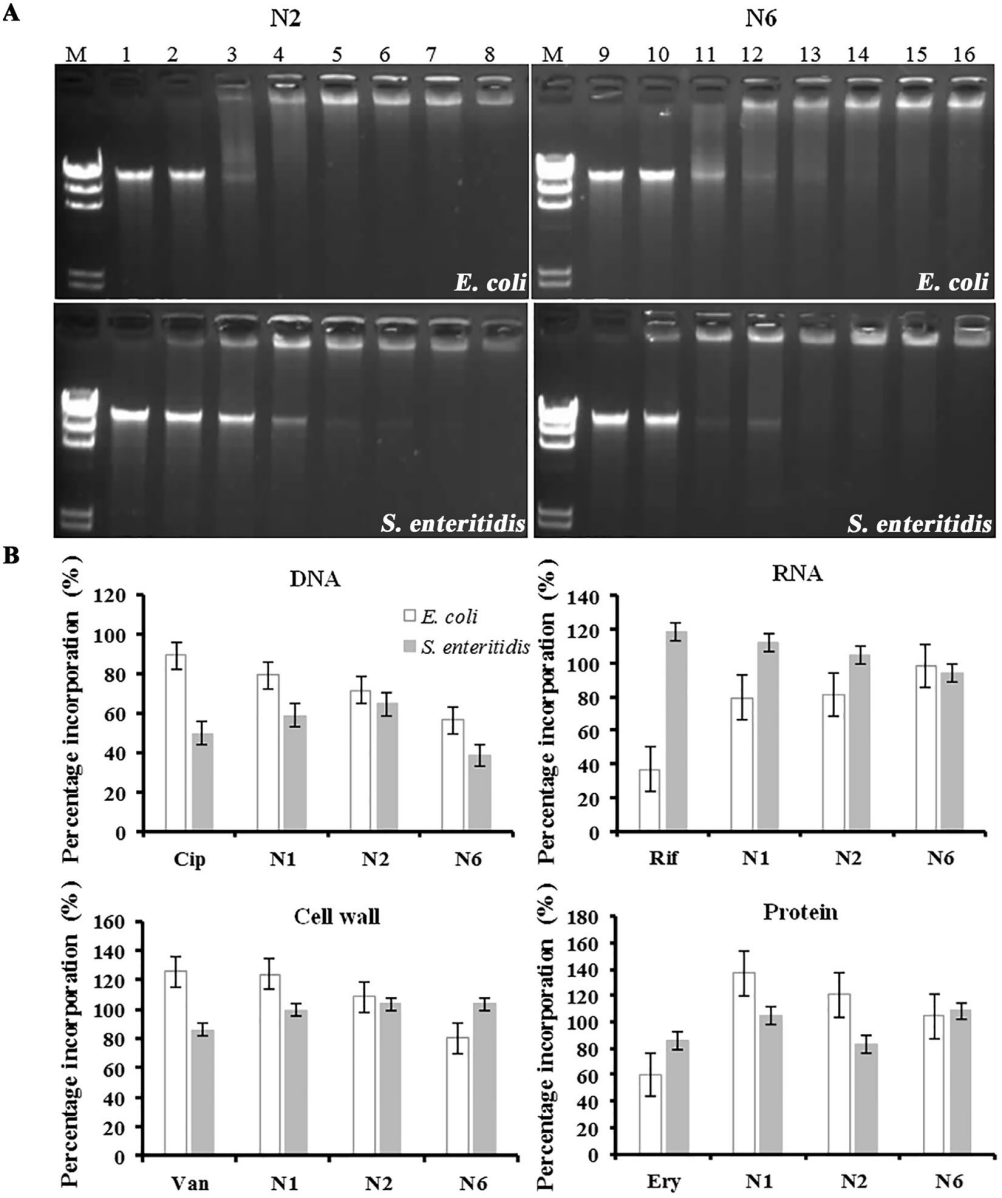
Interaction of N2 and N6 with bacterial DNA and biomacromolecule synthesis. (**A**) Interaction of N2 and N6 with bacterial genomic DNA by a gel migration assay. M: DNA marker λDNA/*Hind*III; 1–8: the mass ratios of N2/ DNA were 0, 0.05, 0.1, 0.15, 0.2, 0.25, 0.3, and 0.4; 9–16: the mass ratios of N6/DNA were 0, 0.05, 0.1, 0.15, 0.2, 0.25, 0.3, and 0.4. (**B**) Impact of N2 and N6 on macromolecular synthesis in *E*. *coli* and *S*. *enteritidis*. Incorporation of 3H-thymidine (DNA), 3H-uridine (RNA), 3H-glucosamine (peptidoglycan), and 3H-leucine (protein) was determined in cells treated with 1× MIC N1, N2, and N6, respectively. Ciprofloxacin (Cip, 8× MIC), rifampicin (Rif, 4× MIC), vancomycin (Van, 2× MIC), and erythromycin (Ery, 2× MIC) were used as controls. The results are given as the mean ± SEM (n = 3).

In an attempt to clarify the molecular mechanism of action, the DNA-binding properties of N2 and N6 were examined by analyzing electrophoretic mobility of DNA bands at various mass ratios of peptides to DNA on an agarose gel. N2 and N6 inhibited the migration of genomic DNA from *E*. *coli* at mass ratios greater than 0.1 and 0.15 (Fig. 5A), respectively. The migration of genomic DNA from *S*. *enteritidis* was suppressed by N2 and N6 at mass ratios greater than 0.2 and 0.15, respectively. This result indicated that N2 and N6 can bind to bacterial DNA.

#### Cell cycle arrest at the I-/R-phase

Changes in DNA frequency distribution histograms of bacterial cells exposed to N2 and N6 for different times are shown in Supplementary Table S3 and Supplementary Figs S7 and S8. With incubation of *E*. *coli* cells with N2 and N6 for 0.5 h, the proportion of cells in I-phase appeared to increase from 33.4 to 41.9% and to 39.4%, respectively. The population of I-phase cells increased to 50.6% and 46.5% with incubation for 2 h in the presence of N2 and N6, respectively. In contrast, decreases in the percentages of cells in the R- and D-phases were observed. This indicated that N2 and N6 could induce *E*. *coli* cell cycle arrest in the I-phase. Exposure to N2 and N6 for 5 min−2 h in *S*. *enteritidis* cells led to an increase in the percentage of R-phase cells in a time-dependent manner (from 65.0% to 80.6% and from 66.8% to 82.3%, respectively) and a corresponding reduction in the percentage of cells in the I-phase (from 26.0% to 15.8% and from 15.8% to 5.2%, respectively) and D-phase (from 8.0% to 3.6% and 17.4% to 5.5%, respectively). This indicated that N2 and N6 induced cell cycle arrest in the R-phase in *S*. *enteritidis* cells.

#### Inhibition of DNA and protein synthesis

The incorporation of radioactive precursors into DNA (^3^H-thymidine), RNA (^3^H-uridine), protein (^3^H-leucine), and peptidoglycan (^3^H-glucosamine) was measured to evaluate the effects of N2 and N6 on macromolecular synthesis in *E*. *coli* CVCC195 and *S*. *enteritidis* CVCC3377 cells. As shown in Fig. 5B, a significant inhibition of ^3^H-thymidine (28.6%) and ^3^H-uridine (18.7%) incorporation was observed with exposure of *E*. *coli* cells to 1× MIC N2 for 15 min, suggesting that N2 is a DNA and RNA synthesis inhibitor. N6 induced a decrease in DNA (43.8%) and cell wall (19.6%) synthesis, suggesting that N6 is a DNA and cell wall synthesis inhibitor. In *S*. *enteritidis*, however, N2 caused an inhibition in the incorporation of radioactive precursors into DNA (35.5%) and protein (17.0%), and N6 inhibited DNA (61.8%) and RNA (5.7%) biosynthesis.

### N2/N6 bound to LPS and protected mice from lethal challenge with LPS

#### Harboring predicted LPS-binding sites

**Figure 6:**
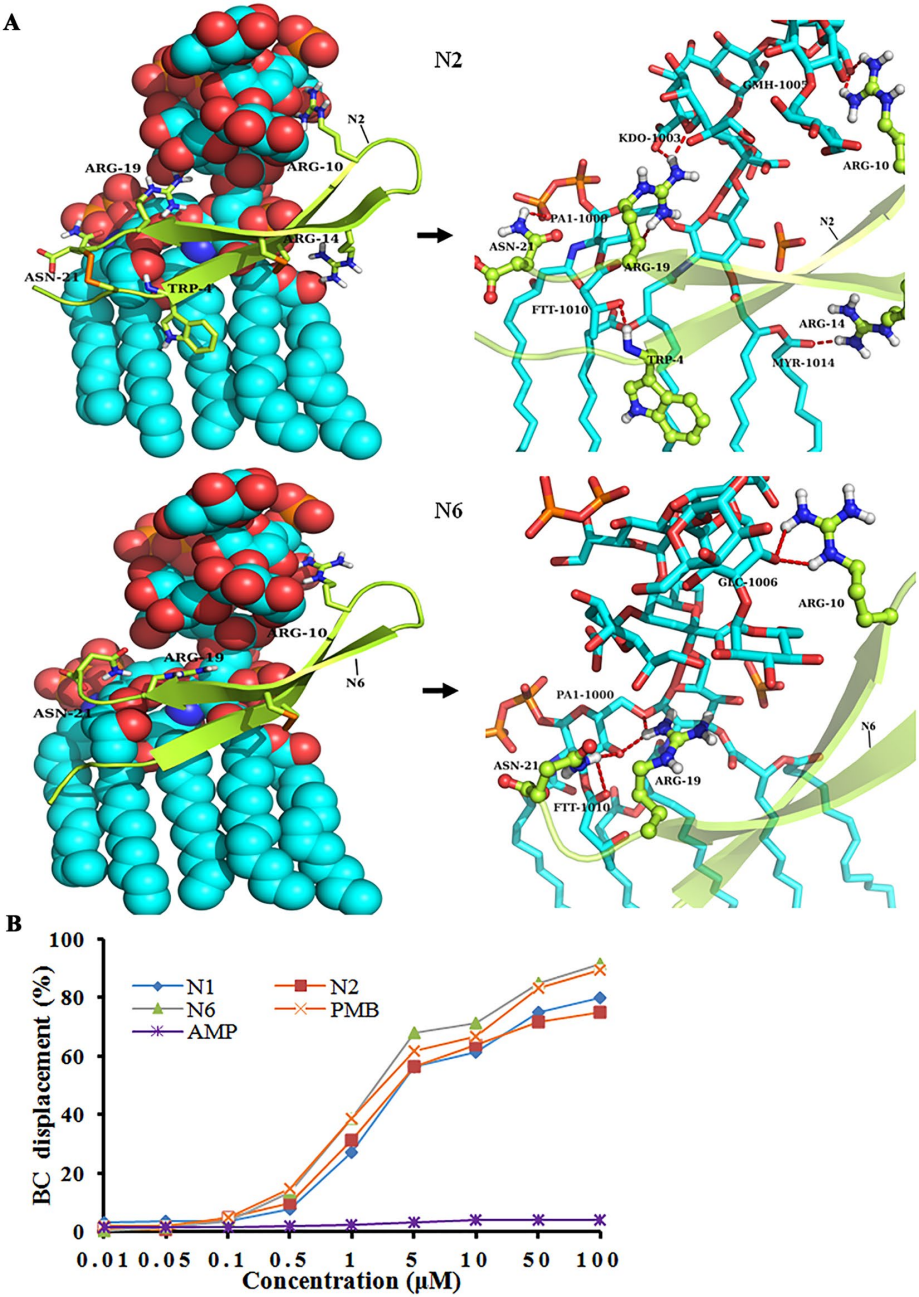
Potential docking of N2 and N6 with LPS. (**A**) The complex structure of N2 and N6 and LPS (left) and detailed view of the binding pocket (right). N2 and N6 are shown as cartoons and the key residues (N2: Trp4, Arg10, Arg14, Arg19, and Asn21; N6: Arg10, Arg19, and Asn21) are shown as sticks and balls. In this proposed autodock-generated model, the fatty acid chains FTT-1010, 63-α-D-glucosyl-maltotriose GMH-1005, MYR-1014, PA1-1000, and the monophosphate group that belongs to the lipid A moiety form hydrogen bonds and salt bridges to Trp4, Arg10, Arg14, and Asn21 of N2, while Arg19 forms a hydrogen bond with the 2-Keto-3-deoxyoctonate (KDO-1003), which belongs to the core polysaccharide of LPS. The fatty acid chain FTT-1010, glucose GLC-1006, and PA1-1000, respectively, form hydrogen bonds to Arg10, Arg19, and Asn21 of N6. (**B**) Binding affinity of N2 and N6 to LPS. Ampicillin (AMP) and PMB were used as negative and positive controls, respectively.

3D-structures of N1, N2, and N6 peptides were docked onto LPS to obtain models of the peptide/LPS complexes (Fig. 6A). Interactions between N1 and LPS were presumably stabilized by hydrogen bonding between positively charged side chains of the His9, Arg14, and Arg19 basic residues and the fatty acid chains MYR-1014, GLC-1007, and KDO-1003 of LPS (Fig. 6A). The Trp4, Arg10, Arg14, and Asn21 residues in N2 appear to form a number of salt-bridges and hydrogen bonds with the fatty acid chains FTT-1010, 63-α-D-glucosyl-maltotriose GMH-1005, MYR-1014, PA1-1000 and the monophosphate group of the lipid A moiety of LPS. Arg19 forms a hydrogen bond with the 2-Keto-3-deoxyoctonate (KDO-1003), which belongs to the core polysaccharide of LPS (Fig. 6A). The N6-LPS docked complex reveals probable hydrogen bonds or salt bridges between side chains of the Arg10/Arg19/Asn21 residues and FTT-1010/glucose GLC-1006/PA1-1000 of LPS (Fig. 6A). This indicated that N1, N2, and N6 have different LPS-binding sites and different modes of action.

#### LPS binding affinity in vitro

To reveal the mechanism of N1 analogues, binding to LPS was determined using the BODIPY’-TR-cadaverine (BC) displacement assay. PMB, a positive control^33^, showed potent LPS binding affinity (Fig. 6B), whereas ampicillin did not displace BC and bind to LPS (Fig. 6B). Similarly to PMB, N1, N2, and N6 induced a significant dose-dependent probe displacement, reflecting their high capacity to bind to LPS. The BC displacement percentage of N6 (91.5%) at 100 μM was greater than that of N1 (79.8%), N2 (75.0%), and PMB (89.3%), indicating that N6 has the highest binding affinity to LPS.

### N2/N6 protected mice from lethal challenge with *E*. *coli and S*. *enteritidis* or LPS

#### *E*. *coli*-induced peritonitis

**Figure 7:**
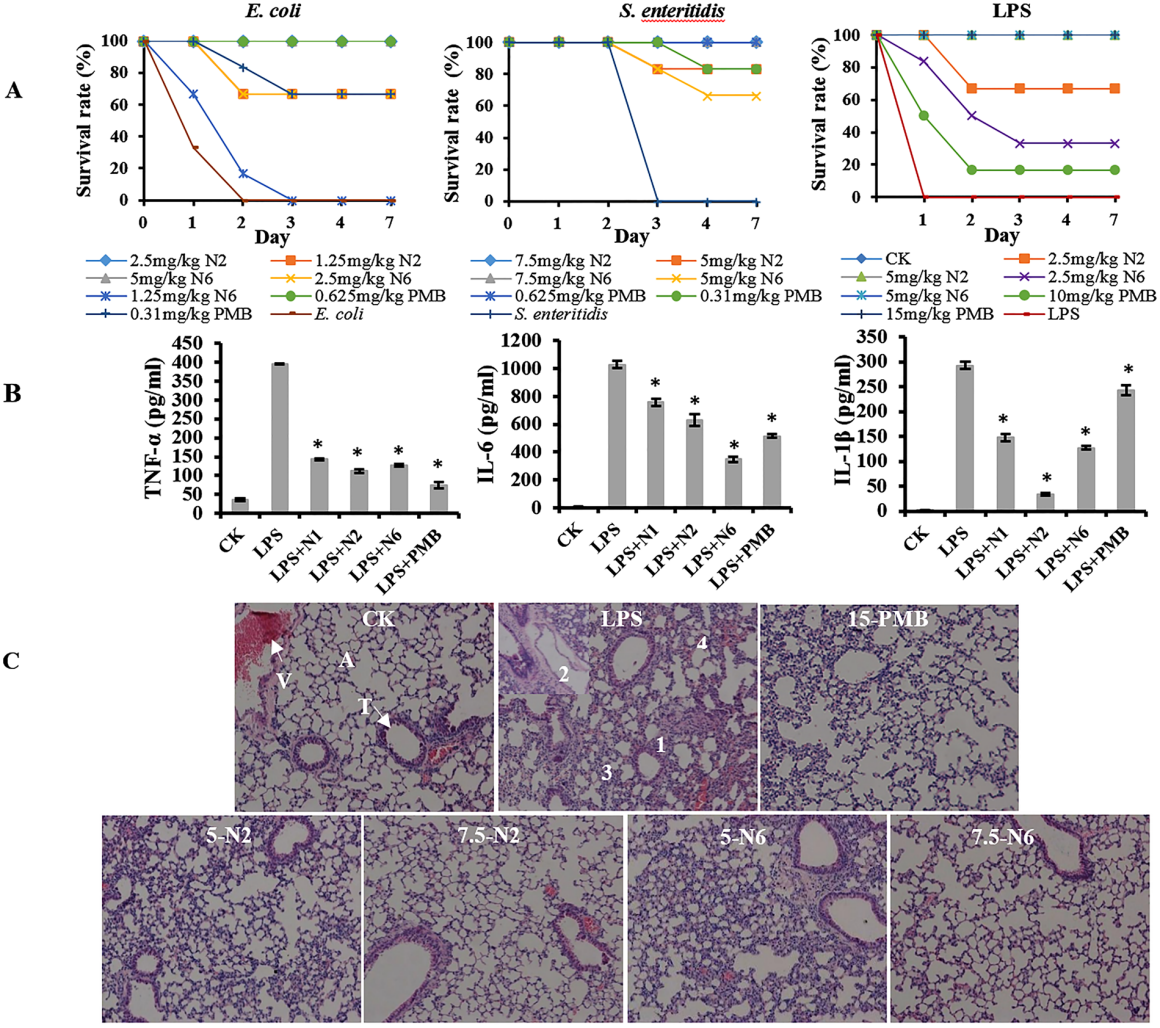
Effect of N2 and N6 on bacteria-induced and LPS-induced responses *in vivo*. (**A**) Survival of mice treated with N2 and N6 in *E*. *coli*, *S*. *enteritidis*, and LPS lethal models. Mice were intraperitoneally injected with *E*. *coli* (2.5 × 10^8^ CFU/ml), *S*. *enteritidis* (1 × 10^6^ CFU/ml), and LPS (30 mg/kg of body weight), respectively, followed by injection with N2, N6 or PMB at 0.5 and 8 h. Survival was recorded for 7 days. (**B**) Inhibition of LPS-induced cytokine release in C57BL/6 mice by N2 and N6. The results are given as the mean ± SEM. Differences between groups were determined by one-way ANOVA followed by SPSS analysis (n = 3 per group). *p < 0.05 compared to the LPS-treated group. (**C**) Protection of lungs from damage by LPS. Lung specimens were collected from sacrificed mice after LPS injection and treatment. The sections were stained with hematoxylin and eosin. V: vessel; A: alveoli; T: trachea. 1: alveolar septum thickening; 2: pulmonary alveolar collapse; 3: inflammatory cell infiltration.

To evaluate the therapeutic activity of N2 and N6 in a peritonitis model, mice were intraperitoneally inoculated with *E*. *coli* CVCC1515 and *S*. *enteritidis* CICC22956 strains, respectively. Mice in the uninoculated control group survived throughout the experimental period (Fig. 7A). The untreated mice began to die 8 h after inoculation with *E*. *coli*, and all untreated mice died within 48 h. After treatment with 1.25 and 2.5 mg/kg N2, the survival rates of mice were 66.7% and 100%, respectively, which was higher than for N6 at the same doses (16.7% and 66.7%). The survival rate of mice treated with 5 mg/kg N6 was 100%. The survival rates of mice treated with 0.31 and 0.625 mg/kg PMB were 66.7% and 100%, respectively (Fig. 7A).

#### *S*. *enteritidis*-*induced* peritonitis

In another peritonitis model, mice were injected with N2/N6 (5 mg/kg and 7.5 mg/kg) or PMB (0.31 and 0.625 mg/kg) at 0.5 h and 8 h after challenge with *S*. *enteritidis*. Mice in the uninoculated control group survived throughout the experimental period (Fig. 7A). All mice without treatment died within 72 h after inoculation with *S*. *enteritidis*. After treatment with 5 mg/kg N2 and N6, the survival rates of mice were 83.3% and 66.7%, respectively. All mice treated with 7.5 mg/kg N2 or N6 survived. The survival rates of mice treated with 0.31 and 0.625 mg/kg PMB were 83.3% and 100%, respectively (Fig. 7A).

#### LPS-induced endotoxemia

The LPS-detoxifying activity of N2 and N6 was evaluated in mice challenged with LPS. At 0.5 h and 8 h after challenge with LPS, mice were treated with N2/N6 (2.5 and 5 mg/kg) or PMB (10 and 15 mg/kg). Mice in an unchallenged control group did not die during the experimental period (Fig. 7A). Mice without treatment began to die at 8 h after challenge with LPS, and all untreated mice were dead within 24 h. After treatment with 2.5 mg/kg N2 and N6, the survival rates of mice were 66.7% and 33.3%, respectively. Meanwhile, mice administered 5 mg/kg N2 and N6 improved their survival rates to 100%. The survival rates of mice treated with 10 and 15 mg/kg PMB were 16.7% and 100%, respectively (Fig. 7A). This result indicated that both N2 and N6 can protect mice from lethal LPS challenge *in vivo*, and have better therapeutic efficacy than PMB.

#### Inhibition of inflammatory cytokine production

To further explore the effects of N2/N6 on inflammatory cytokines, an endotoxemia mouse model was constructed by intraperitoneal injection with 10 mg/kg LPS followed by treatment with peptides or antibiotics. As shown in Fig. 7B, similarly to PMB, N2 and N6 significantly reduced the release of tumor necrosis factor-α (TNF-α), interleukin-6 (IL-6), and interleukin-1β (IL-1β). The levels of TNF-α, IL-6, and IL-1β in endotoxemia mice treated with N2 (111.9, 630.3, and 33.3 pg/ml, respectively), N6 (127.2, 350.1, and 127.2 pg/ml, respectively) or PMB (74.0, 514.9, and 242.8 pg/ml, respectively) were significantly lower than those of the corresponding LPS-challenged control groups (394.9, 1026.8, and 292.9 pg/ml, respectively). This indicated that both N2 and N6 can inhibit the production of proinflammatory cytokines.

#### Protection of LPS-induced lung injury

To explore if N2 and N6 can protect mice from LPS-induced lung injury, lung tissues were dissected and examined 7 d after treatment. As shown in Fig. 7C, no pathological change was observed in the lungs of mice injected only with PBS, whereas acute injury, including fibrosis, alveolar septum distention, inflammatory cells, and red blood cell infiltration, was found in the untreated LPS-challenged mice. In contrast, after treatment with 5 mg/kg N2 and N6, there was less apparent damage in the lungs of treated mice. At a high dose of 7.5 mg/kg N2 or N6, no obvious pathological changes were found in the murine lung tissues. The efficacy of N2 and N6 was higher than that of PMB. These data demonstrated that both N2 and N6 may be potential therapeutics for *E*. *coli* and *S*. *enteritidis* infection and LPS-induced endotoxemia.

## Discussion

In this work, a series of N1 analogues were designed by changing disulfide bonds, hydrophobicity, or charge to reduce cell cytotoxicity, investigate the effects of disulfide bonds on antimicrobial activity, and develop novel cell-selective β-sheet AMPs with improved antimicrobial activity. Among these analogues, the “rocket” analogue N2, with two disulfide bonds, and the “kite” analogue N6, with one disulfide bond, were found to be the most active against both *E*. *coli* and *S*. *enteritidis* (Table 2), and multiple levels of selective antimicrobial mechanisms of the two peptides and detoxifying activity were systematically investigated *in vitro* and *in vivo*.

It has been demonstrated that an increase in the hydrophobic moment and net charge leads to an increase in antibacterial activity of AMPs^34^. In our study, the favorable effect of residue replacement on the activity of N2 may result from increased hydrophobicity due to the replacement of the Gly residue at positions 1 and 12 in N1 by Ala (Table 2; Supplementary Fig. S3). On the one hand, the Gly → Ala substitution resulted in a conformational perturbation of approximately 9.5% of the α-helical structure formation (Table 1, Fig. 2) due to the introduction of a β-carbon into the 1 and 12 positions of N2^35^, which controls the peptide flexibility and contributes to optimal antimicrobial activity (Table 2)^36^. On the other hand, the Gly → Ala substitution led to a marked decrease in the thermal stability of N2 at 100 °C (Fig. 1C), indicating an increase in free energy from the Ala substitution, as observed or other peptides^35, 36^. Additionally, compared to N1, a more positively charged N-terminus (from +4 to +5) of the “rocket” N3 analogue, through Lys replacement at position Trp4, decreased activity (Table 2), while the substitution of Asn at position 5 by Ala led to an increase in hydrophobicity (from −0.243 to −0.113) (Table 1). This may be attributed to destabilization of the pore due to increased repulsion among peptide monomers or to an imbalance between positively charged amino acids and hydrophobicity^37, 38^, although the correlation with activity is not fully clarified. Introduction of Trp at the N-terminus of N1 gave rise to the “rocket” N4 analogue with a potent hydrophobic nature (from −0.243 to −0.273) which showed relatively weaker antimicrobial activity than N1, indicating that the Trp1 side chain is unimportant for activity. Linear N5 and N8 analogues exhibited the lowest hydrophobicity (−0.888 and −0.376) and antibacterial activity (Tables 1, 2). Therefore, these results demonstrated that hydrophobicity is very important for the full activity of N1 peptides.

Due to the small size of AMPs, it is thought that disulfide bonds are critical for maintaining the 3D-folding and structure of the molecule. However, several AMPs, such as hBD3 and μ-conotoxins, have shown that disulfide bonds are not absolutely necessary for antimicrobial activity against pathogens^20, 22^. Furthermore, variants of HNP-1 and cryptdin-4 with fewer disulfide bonds are more antimicrobial than the wild-type peptide against *E*. *coli*, *S*. *aureus*, and *P*. *aeruginosa* ^19, 24^. In this study, the antimicrobial activity of the “kite” analogue N6 was almost unaffected by the deletion of the first disulfide bond (Table 2), indicating that the first disulfide bond (Cys3-Cys20) in N1 is not required for its antimicrobial activity. However, the second disulfide bond (Cys7-Cys16) in the “bullet” analogue N7 is indispensable for its antimicrobial activity. Linear N5 and N8 analogues without disulfide bonds lost considerable activity against all tested bacterial strains. These data suggest that the second disulfide bond (Cys7-Cys16), which is proximal to the β-sheet, plays a major role in stabilizing the β-sheet structure within N1 and maintaining the antibacterial activity, in contrast to the PG-1 variants^27^. Perhaps this is related to differences in the nature, the structure, and the spacing between their disulfide bonds.

Our previous study has demonstrated that N1 can interact electrostatically with the cell membrane of *E*. *coli*, permeabilize the outer and inner membrane, cause depolarization and leakage of contents, and eventually lead to cell death^30^. In this study, N2 and N6 exhibited remarkable specificity for particular AMP–bacteria pairings. Both N2 and N6 more strongly permeabilized a model bacterial membrane than did N1, the outer and inner membrane of *E*. *coli*, and the outer but not the inner membrane of *S*. *enteritidis* (Fig. 3C). This may be related to more conformational flexibility in the N- and C-terminal regions of N2 and N6 (Fig. 2B), additionally conferring less toxicity than N1 (Fig. 1A and B), which is consistent with other AMPs such as LAK120, LAK80 and their analogues^39^. Furthermore, the inner membrane potential of *S*. *enteritidis* is rarely changed by the two peptides and more than 99% of the inner membrane integrity was maintained (Supplementary Fig. S6C). This is consistent with a previous report in which pleurocidin and its analogues did not cause inner membrane permeabilization despite inhibiting intracellular macromolecular synthesis in *E*. *coli* ^40^. Moreover, we demonstrated that the activity of the two peptides against *S*. *enteritidis* cells was associated with the accumulation of ROS, which is consistent with the results of a previous study which found that ROS accumulation induced by N1 may play an important role in apoptosis^30^. These results suggest that the nature of AMPs and the characteristics of the bacterial cell membrane determine the antimicrobial actions of AMPs^41^.

Another significant effect of N2 and N6 on *E*. *coli* and *S*. *enteritidis* was evident from their DNA-binding properties. The migration of genomic DNA from *E*. *coli* and *S*. *enteritidis* was inhibited by N2 and N6 when a DNA mass ratio was greater than 0.1 (Fig. 5A), which is similar to our previous report that N1 bound tightly to DNA by inserting base pairs and changing the DNA conformation^30^.

The cell wall is a shape-maintaining element in bacteria^42^. The collapse of *E*. *coli* cells treated with N6 was observed in SEM images (Fig. 4A), which seems to be a consequence of cell wall synthesis inhibition by N6 (Fig. 5B). Additionally, both N2 and N6 inhibited other macromolecular synthesis in bacterial cells (Fig. 5B). The inhibition of DNA synthesis in *E*. *coli* cells was accompanied by slightly delayed cessation of RNA (N2) and cell wall (N6) synthesis (Fig. 5B). This effect of N2 on *E*. *coli* was similar to that observed for N1 (30). However, synthesis of DNA, RNA, and proteins in *S*. *enteritidis* treated with N2 and N6 seems to be affected in a direct way (Fig. 5B) but probably by different mechanisms, as confirmed by SEM images showing intact cell morphology (Fig. 4A). This is consistent with recently published data for pyrrhocoricin and oncocin which function by repressing translation or by binding to the 70S ribosome^43, 44^.

LPS, a highly conserved major component of the outer membrane of gram-negative bacteria, is released spontaneously and rapidly during bacterial growth and after exposure to antibiotics^7, 45^. It is the primary trigger of endotoxemia because it stimulates monocytes and macrophages to produce large amounts of proinflammatory mediators, such as TNF-α and IL-6^7, 46, 47^. Dixon *et al*. reported that the synthetic AMP KSL-W can bind to the LPS of *E*. *coli* and various oral bacteria^14^. Our previous study has demonstrated that N1 can bind LPS and reduce the release of proinflammatory cytokines in LPS-induced acute peritonitis in mice^30^. In this study, BC probe displacement and docking calculation confirmed binding of N2 and N6 with LPS through the formation of hydrogen bonds and salt bridges (Fig. 6 and Supplementary Fig. S9). It is speculated that the binding of N2 and N6 with LPS may initially interrupt the interaction of LPS with its toll-like receptor, and subsequently inhibit the release of LPS-induced IL-6, IL-1β and TNF-α in mice, thereby effectively protecting mice from acute lung injury (Fig. 7B and C). Moreover, N2 and N6 enhanced the survival rates of peritonitis- and endotoxemia-induced mice, which were significantly superior to PMB in the endotoxemia model (Fig. 7A). Together, these results suggest that, similarly to N1, both N2 and N6, having low cytotoxicity and no antimicrobial resistance, are potential antibacterial and endotoxemia therapeutics.

In this study, we designed a series of N1 analogues based on disulfide bonds, hydrophobicity, and charge. Both N2 and N6 showed potent antimicrobial activity against *E*. *coli* and *S*. *enteritidis*, with N2 having higher activity than N1. N2 and N6 had lower hemolysis and cytotoxic effects than N1 due to increased flexibility in the N- and C-terminal regions. Both N2 and N6 disrupted the outer and inner membranes of *E*. *coli*, induced cell cycle arrest at the I-phase, and inhibited DNA/RNA/cell wall synthesis in *E*. *coli*. For *S*. *enteritidis*, the outer membrane rather than the inner membrane was permeabilized, cell cycle was arrested at the R-phase, and DNA/RNA/protein synthesis was inhibited by N2 and N6. N2 and N6 protected mice from mortality induced by bacterial infection (*E*. *coli* and *S*. *enteritidis*) and *E*. *coli* LPS. Our findings suggest that N2 and N6 are promising candidates for further research as new antimicrobial and anti-endotoxin agents.

## Materials and Methods

### Peptide design

The N1 analogues were designed based on the replacement of Gly, Cys and Gln residues by the hydrophobic/cationic residues Ala/Lys and the removal of disulfide bonds or the addition of a hydrophobic Trp residue at positions 1, 3, 4, 5, 7, 12, 16, and 20 (Table 1). The physicochemical properties of these peptides were calculated with Protparam (http://web.expasy.org/protparam/) and APD3, Antimicrobial Peptide Calculator and Predictor (http://aps.unmc.edu/AP/prediction/prediction_main.php). Three dimensional (3D) molecular structures of N1 and its analogues were made using the PyMOL molecular modeling system (http://www.pymol.org/). All peptides with 90% purity were synthesized by the ChinaPeptides Co. Ltd. (Shanghai, China).

### Antimicrobial activity

The antimicrobial activity of peptides was determined by the microbroth dilution method^48^. The bacterial strains were incubated in Mueller-Hinton (MH) medium at 37 °C overnight until the mid-log phase (OD_600 nm_ reading of 0.5). Bacteria were diluted to 1 × 10^5^ CFU/ml and added into 96-well plates, followed by the addition of serial dilutions of peptides (from 0.0625 to 128 μg/ml). The plates were incubated at 37 °C for 18–24 h. The MIC value was determined as the lowest peptide concentration at which no bacterial growth was observed. All tests were conducted in triplicate.

### Hemolysis

The hemolytic activity of N2 and N6 was evaluated by determining the amount of hemoglobin that was released from fresh mouse erythrocytes^29^. The blood cells were washed three times with 10 mM PBS (pH 7.4) and centrifuged at 1,500 rpm for 10 min at room temperature. In brief, 100 μl of erythrocyte solution (8%, v/v) was mixed with 100 μl of a series of peptide solutions and incubated for 1 h at 37 °C. The mixture was then centrifuged at 1,500 rpm for 5 min, and the absorbance of supernatants was measured at 540 nm. Values of 0 and 100% hemolysis were determined in PBS and 0.1% Triton X-100, respectively. Three replicates were performed for each condition. Hemolysis (%) = [Abs_540 nm_ of the treated sample − Abs_540 nm_ of the negative control)/(Abs_540 nm_ of positive control − Abs_540 nm_ of negative control)] × 100.

### Cytotoxicity

The colorimetric MTT assay was used to determine the effect of peptides on the viability of murine peritoneal RAW264.7 macrophage cells^49^. Cells (5 × 10^3^ cells/well) were added into 96-well microtiter plates and incubated in a humidified 5% (v/v) CO_2_/air environment at 37 °C for 24 h. A series of peptide solutions were added and incubated for 48 h. Untreated cells were used as controls. The MTT solution was added into plates, incubated for 4 h, and then removed from plates. Dimethyl sulfoxide (DMSO) was added into plates and the absorbance was measured at 570 nm with a spectrophotometer. The rate of inhibition of cell proliferation was calculated using the following formula: Growth inhibition (%) = (Abs_570 nm_ of control − Abs_570 nm_ of treated sample)/Abs_570 nm_ of control × 100.

### Antimicrobial resistance

Antimicrobial resistance of N2 and N6 was measured by the sequential passaging method^50^. *E*. *coli* cultures (10^6^ CFU/ml) were treated with N2 and N6 (from 0.25× to 8× MIC), respectively. At 24 h intervals, cultures from the second-highest concentrations (OD ≥ 0.5) were diluted 1:100 into fresh MH broth containing N2 or N6. This serial passaging was repeated daily for 18 days. Any cultures that grew at higher than the MIC levels were passaged on peptide-free MH agar plates and the MIC was then determined by broth microdilution. This was repeated three times.

### Sensitivity of N2/N6 to temperature, pH, and proteases

To determine effects of temperature on antibacterial activity, N2 and N6 were incubated for 1 h at 4, 20, 40, 60, 80 and 100 °C in PBS. The pH stability of N2 and N6 was determined after 3 h incubation in 100 mM glycine-HCl buffer (pH 2.0), sodium acetate buffer (pH 4.0), sodium phosphate buffer (pH 6.0), Tris-HCl buffer (pH 8.0), or glycine-NaOH buffer (pH 10.0). In addition, N2 and N6 were incubated for 4 h at 37 °C in pepsin (3000 U/mg, pH 2.0), papain (500 U/mg, pH 6.0), and trypsin (250 U/mg, pH 8.0) (10:1, w/w) solutions, respectively. The antibacterial activity of peptides was estimated against *E*. *coli* CVCC195 and *S*. *enteritidis* CVCC3377 cells by a microdilution plate assay^48^. All tests were conducted in triplicate.

### CD and NMR analysis of N2/N6

The secondary structure of peptides was investigated in ddH_2_O and SDS solutions by CD spectroscopy^51^. The CD spectra of N2 and N6 were measured via a MOS-450 spectropolarimeter (Bio-Logic, Grenoble, France) using a 1.0 mm path-length cuvette. Peptides were dissolved in ddH_2_O or SDS solutions (10–40 mM). The spectra of peptides were recorded from 180 to 260 nm at 25 °C at a scanning speed of 100 nm/min with a step resolution of 2.0 nm and an integration time of 2 s. Data were analyzed using CDNN software.

The structures of N1, N2 and N6 in 90% H_2_O/10% D_2_O were determined by solution state NMR. All NMR experiments were acquired at 298 K on an Agilent DD2 600 MHz spectrometer equipped with a cold-probe. For each peptide, approximately 5 mg of solid powder was dissolved in 500 µl aqueous solvent (10% D_2_O, 90% H_2_O, v/v) to make the NMR samples. A series of 1D and 2D spectra, including ^1^H, ^1^H-^1^H TOCSY, ^1^H-^1^H NOESY, ^1^H-^13^C HSQC, and ^1^H-^15^N HSQC, were acquired for the structure elucidation. Amide proton temperature coefficients were derived from four TOCSY experiments at different temperatures (293, 298, 303 and 308 K) for investigating intramolecular hydrogen bonds. All TOCSY experiments were carried out using an 80 ms MLEV-17 spin-lock with a field strength of 6839 Hz. The mixing time for each NOESY experiment was 150 ms. The spectra widths were 7396.4 * 7396.4 Hz for the ^1^H-^1^H, 7396.4 * 21124.9 Hz for the ^1^H-^13^C and 7396.4 * 1945.6 Hz for the ^1^H-^15^N correlation spectra. 256, 140 and 128 complex points were collected for the indirect proton, carbon and nitrogen dimensions, respectively, and 512 complex points were collected for the directly observing dimension. The chemical shifts were referenced to DSS at 0.00 ppm for the proton and indirectly for the carbon and nitrogen dimensions^52^. All NMR data were processed using NMRPipe and analyzed with the CcpNmr program suite^53^. Signal assignment was obtained by following the method developed by Wuthrich, and inter-proton distance restraints were derived from the NOESY spectrum. Structure calculation and NOE assignment were performed simultaneously by using the programs CNS and ARIA2^54, 55^. Hydrogen bonding restraints were generated by analyzing the NOE patterns, backbone secondary chemical shifts as well as the amide proton temperature coefficients. Backbone dihedral angle restraints (Φ and Ψ angles) were derived using the program DANGLE incorporated in the CcpNmr suite^56^. A total of 100 structures were calculated, and the 20 structures with the lowest total energy were selected to perform a refinement procedure in water. The protein structure ensemble was displayed and analyzed with the software Pymol (http://www.pymol.org/).

### Effects of N2/N6 on model membranes, cell membranes, and membrane potential

#### Dye leakage experiments from model membranes

Different lipids (Avanti Polar Linpids, Inc.) were used to prepare model membranes composed of POPC/ POPG (4:1 molar ratio) and POPC/cholesterol (1:1) by the extrusion method as previously described^57^. POPC/POPG and POPC/cholesterol LUVs were used to mimic gram-negative bacterial and eukaryotic membranes, respectively. Lipid suspensions (500 μM) loaded with calcein (50 μM) were incubated with peptides at 1×, 2×, and 4× MIC against *E*. *coli* cells or 2-fold dilution concentrations (0.25–256 μg/ml). After incubation for 5 min, the fluorescence (F) (λ_excitation _= 492 nm, λ_emission_ = 518 nm) was measured on a microplate reader. The calcein leakage released from LUVs treated with N2 or N6 was determined as % Leakage = (F − F_0_)/(F_t_ − F_0_) × 100, wherein F_t_ is fluorescence released from vesicles treated with 1% Triton X-100 and F_0_ is the fluorescence of each sample at T = 0^58^.

#### Outer membrane permeability assay

The outer membrane permeabilization activity of N2 and N6 was determined using the fluorescent dye NPN assay^59^. Mid-log phase *E*. *coli* CVCC195 and *S*. *enteritidis* CVCC3377 cells were collected by centrifugation and washed twice, then suspended in HEPES buffer (pH 7.4) to an OD_600 nm_ of 0.4. Cell suspensions and NPN solutions (10 μM) were added into 96-well black plates, followed by the addition of peptide solutions (1×, 2×, and 4× MIC). Fluorescence intensity was recorded until no further increase was observed with a microplate reader (excitation/emission, 328/438 nm). The cells treated with PMB and PBS were used as positive and negative controls, respectively.

#### Inner membrane permeability assay

Mid-log phase *E*. *coli* CVCC195 and *S*. *enteritidis* CVCC3377 cells (1 × 10^8^ CFU/ml) were incubated with or without 1× MIC peptide solutions at 37 °C for 5, 30, and 120 min. After washing twice with PBS, cells were then incubated with 50 μg/ml PI for 15 min and analyzed using a FACS Calibur Flow Cytometer (BD, USA)^46^.

#### Membrane potential assay

The effect of peptides on bacterial membrane potentials was measured with the lipophilic fluorescent dye rhodamine-123^60, 61^. Mid-log phase *E*. *coli* CVCC195 and *S*. *enteritidis* CVCC3377 cells were harvested by centrifugation and resuspended in PBS (1 × 10^8^ CFU/ml). Then, 1×, 2×, and 4× MIC N2 or N6 solutions were incubated for 2 h with the above cells at 37 °C. After washing twice with PBS, rhodamine-123 was added and incubated at room temperature for 10 min in the dark. Fluorescence intensity was analyzed using a flow cytometer. Untreated cells were used as a blank control.

#### Intracellular ROS accumulation

Intracellular ROS accumulation was measured using the fluorescent dye DHR-123. *E*. *coli* CVCC195 and *S*. *enteritidis* CVCC3377 cells (1 × 10^8^ CFU/ml) were incubated for 2 h at 37 °C with 1×, 2×, and 4× MIC N2 or N6, washed with PBS and stained with DHR-123 (5 μg/ml). The fluorescence of the dyes was measured using a flow cytometer.

### Electron microscopy

Mid-log phase *E*. *coli* CVCC195 and *S*. *enteritidis* CVCC3377 cells (1 × 10^8^ CFU/ml) were incubated with 4× MIC N2 or N6 for 2 h at 37 °C. The bacterial cells were then centrifuged and washed three times with 0.1 M PBS (pH 7.2) and fixed overnight at 4 °C with 2.5% glutaraldehyde. After washing twice with PBS, the cells were post-fixed with 1% osmium tetroxide (OsO_4_) for 2 h, dehydrated in a graded ethanol series (from 50% to 100%) for 15 min each time, and dried by CO_2_. Gold-palladium was sputtered on samples and observed on a QUANTA200 SEM (FEI, Philips, Netherlands)^58^.

After fixation with 2.5% glutaraldehyde at 4 °C overnight, bacterial cells were post-fixed with 1% OsO_4_ for 1 h and washed three times with PBS. The cells were then dehydrated for 7 min each time with a graded acetone series (50%, 70%, 85%, 95% and 100%). The samples were immersed in mixtures of acetone and resin (3:1, 1:1 and 1:3, respectively) for 40 min each time and in a pure epoxy resin overnight in a dryer. The samples were then embedded in capsules containing embedding medium, polymerized at 45 °C for 3 h and at 65 °C for 24 h, respectively. Thin sections were prepared using an ultramicrotome and stained with 1% uranyl acetate and visualized by a JEM1400 (JEDL, Tokyo, Japan)^59^.

### Effects of N2/N6 on bacterial genomic DNA migration, cell cycle and macromolecular synthesis

#### DNA gel migration assay

The genomic DNA of *S*. *enteritidis* was incubated for 10 min at room temperature with different concentrations of N2 and N6 in binding buffer (5% glycerol, 10 mM Tris-HCl, pH 8.0, 20 mM KCl, 1 mM EDTA, 1 mM dithiothreitol and 50 mg/ml BSA). The mass ratios of peptides and DNA were 0, 0.05, 0.1, 0.15, 0.2, 0.25, 0.3, and 0.4. The migration of DNA was assessed by electrophoresis on a 1% agarose gel^58, 59^.

#### Cell cycle analysis

Mid-log phase *S*. *enteritidis* CVCC3377 cells (1 × 10^8^ CFU/ml) were incubated with 1× MIC at 37 °C for 5 min, 30 min and 120 min^59^. After centrifugation and washing twice with PBS, the cells were fixed in precooled ethanol overnight at 4 °C. The cells were then harvested, washed, and resuspended in PBS. After incubation with RNAse A (0.5 mg/ml) at room temperature for 15 min, the cells were stained with PI solution for 15 min and examined using a flow cytometer.

#### Macromolecular synthesis

Effects of N2 and N6 on DNA, RNA, protein and peptidoglycan synthesis were determined in *S*. *enteritidis* by measuring incorporation of radioactive precursors of L-[methyl-^3^H] thymidine, [5-^3^H] uridine, L-[3,4,5-^3^H] leucine, and D-[6-^3^H(N)] glucosamine hydrochloride^50^. Mid-log phase *S*. *enteritidis* CVCC3377 cells (10^5^ CFU/ml) were incubated with 1× MIC N2, N6 or antibiotics at 37 °C for 15 min. Radioactive precursors were added into cells (1 μCi/ml) and shaken at 37 °C for 20 min. Ice-cold 25% trichloroacetic acid (TCA) was added and placed on ice for 30 min. The cells were centrifuged and washed twice with ice-cold 25% TCA. After drying, the samples were mixed with scintillation fluid and counted for their radioactivity using a MicroBeta 1450 scintillation counter (Perkin Elmer)^62^.

### Docking of N2/N6 with LPS

The NMR structures of N1, N2 and N6 were docked onto LPS by the program AutoDock 4.2^63^. Peptides were used as ligands and backbones were kept rigid, and almost all side chains were defined as flexible. LPS was defined as a receptor and was kept rigid. Grid maps representing LPS were constructed using 70 × 80 × 80 points, with a grid spacing of 0.375 Å and grid center of the H2 atom of the glucosamine II (GlcN II) residue in lipid A. Docking of peptides with LPS was generated by using a Lamarchian genetic algorithm (LGA) with a translation step of 0.2 Å, a quaternion step of 5°, and a torsion step of 5°. The maximum number of energy evaluations increased to 15,000,000. One hundred LGA docking runs were performed.

### LPS binding affinity *in vitro*

The binding affinity of N2 and N6 to the lipid A region of LPS was determined by the fluorescence probe BC displacement method^64^. Equal volumes of 40 μg/ml LPS and 10 μM BC in 50 mM Tris buffer (pH 7.4) were mixed, added into 96-well plates and incubated in the dark for 4 h at room temperature. Different concentrations of N2, N6 or antibiotics were added and kept in the dark for 1 h at room temperature. Fluorescence was then measured using a microplate reader (excitation/emission, 580/620 nm). The occupancy rate was calculated by the following equation: BC displacement rate (%) = (1 − (F_0_ − F)/(F_0_ − F_max_)) × 100, wherein F_0_ is the fluorescence intensity of the solution of BC alone; F_max_ is the fluorescence intensity in the presence of LPS at the saturation concentration, and F is the intensity of LPS:BC mixtures at varying displacer concentrations.

### Efficacy of N2/N6 *in vivo*

Mice were purchased from the Beijing Vital River Laboratory Animal Technology Co. Ltd. and were housed under standard conditions and a 12:12 h light/dark cycle. All animal experiments were performed in accordance with the Animal Care and Use Committee of the Feed Research Institute of the Chinese Academy of Agricultural Sciences (CAAS), and protocols have been reviewed and approved by the Laboratory Animal Ethical Committee and its Inspection of the Feed Research Institute of CAAS (AEC-CAAS-20090609).

#### Bacteria-induced peritonitis models in mice

Six-week-old female ICR mice (approximately 20 g, six mice per group) were intraperitoneally injected with *E*. *coli* CVCC1515 (2.5 × 10^8^ CFU/ml, 1 ml) and *S*. *enteritidis* CICC22956 (1 × 10^6^ CFUF/ml, 1 ml) strains, respectively, followed by intraperitoneal injection with N2 and N6 (1.25, 2.5, 5, and 7.5 mg/kg of body weight, 0.2 ml) or PMB (0.31, 0.625, and 1.25 mg/kg of body weight, 0.2 ml) at 0.5 and 8 h. Mice injected with only bacteria or saline served as negative (untreated) or blank (unchallenged) controls, respectively. Survival of mice was recorded every 12 h and followed for 7 days.

#### LPS-induced endotoxemia model in mice

Eight-week-old SPF male C57BL/6 mice (20 g ± 5 g, six mice per group) were injected intraperitoneally with *E*. *coli* 0111:B4 LPS (30 mg/kg body weight, Sigma). The mice were injected intraperitoneally with 0.2 ml of N2, N6 (2.5 and 5 mg/kg) or PMB (10 and 15 mg/kg) at 0.5 h and 8 h after challenge with LPS. Mice injected with PBS were used as the negative control. The mouse survival rate was recorded for 7 days.

Nine mice per group were injected intraperitoneally with N2 and N6 (5 mg/kg) or PMB (15 mg/kg) 0.5 h after challenge with 10 mg/kg LPS. Blood was collected in a heparin sodium tube at 2 h or 8 h after LPS injection and incubated for 30 min at 37 °C and then overnight at 4 °C. After centrifugation at 3,000 rpm for 10 min at 4 °C, serum was collected from supernatant. The cytokine levels of TNF-α, IL-6 and IL-1β were detected by the Jiaxuan Biotech. Co. Ltd. (Beijing, China) using an enzyme linked immunosorbent assay (ELISA).

The mice were injected intraperitoneally with N2 and N6 (5 and 7.5 mg/kg) or PMB (15 mg/kg) at 0.5 h and 8 h after challenge with LPS (30 mg/kg). At 7 d after LPS injection, lungs were dissected out and removed, washed with PBS, and fixed in 4% paraformaldehyde at 4 °C for 24 h. The lung tissues were then washed with PBS, dehydrated with a graded ethanol series (75–95%), infiltrated with xylene, and embedded in paraffin wax. Sections were cut, stained with hematoxylin and eosin, and observed by a light microscope. Mice injected with only LPS or PBS served as a negative or blank controls.

### Statistical analysis

All data are presented as the mean ± SEM (standard error of the mean). Comparisons among multiple groups were performed by one-way ANOVA and SPSS analysis. A p-value of <0.05 was considered statistically significant.

## Electronic supplementary material

Supplementary information
