# Supplementary material for: Antibacterial and detoxifying activity of NZ17074 analogues with multi-layers of selective antimicrobial actions against Escherichia coli and Salmonella enteritidis
Source: Sci Rep. 2017 Jun 13;7:3392. doi: 10.1038/s41598-017-03664-2 (PMC5469750; doi:10.1038/s41598-017-03664-2)
Supplement: Supplementary file 1 — Supplementary information [file 41598_2017_3664_MOESM1_ESM.pdf]

Antibacterial and detoxifying activity of NZ17074 analogues with multi-layers of selective antimicrobial actions against *Escherichia coli* and *Salmonella enteritidis*

Na Yang[1, 2*], Xuehui Liu[3*], Da Teng[1, 2*], Zhanzhan Li[1, 2], Xiumin Wang[1, 2], Ruoyu Mao[1, 2], Xiao Wang[1, 2], Ya Hao[1, 2] & Jianhua Wang[1, 2**]**

SUPPORTING IFORMATION

Supplementary 1: Figures

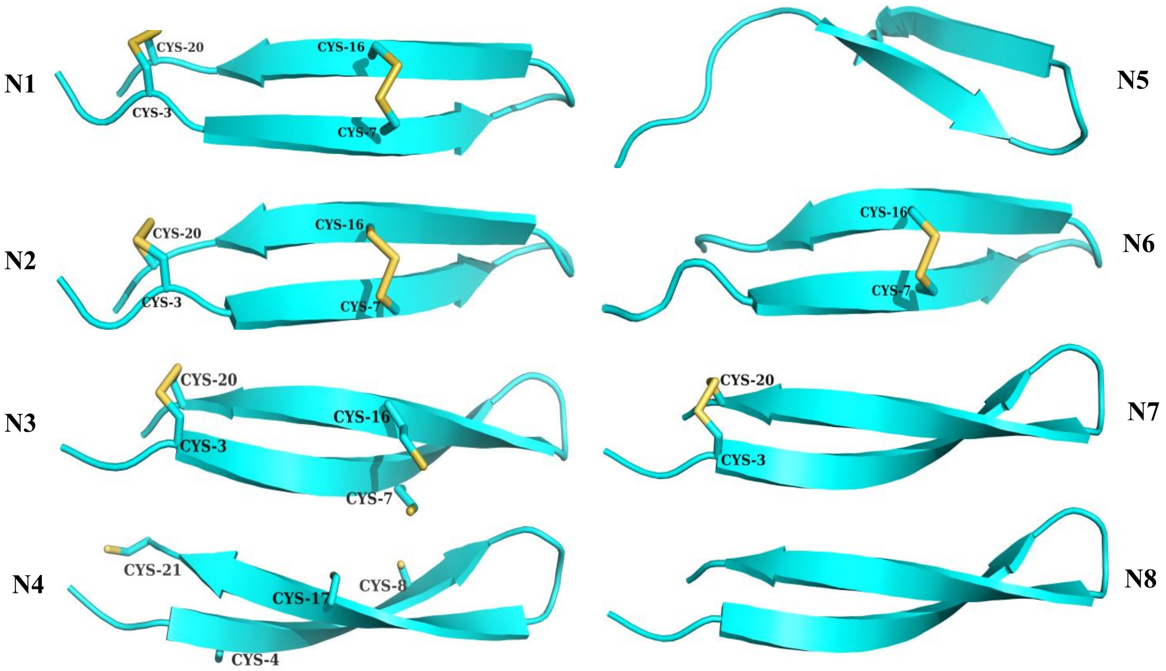

Figure 1 The cartoon representation of N1 and its analogues was generated using PyMOL. Disulfide bridges are shown as sticks.

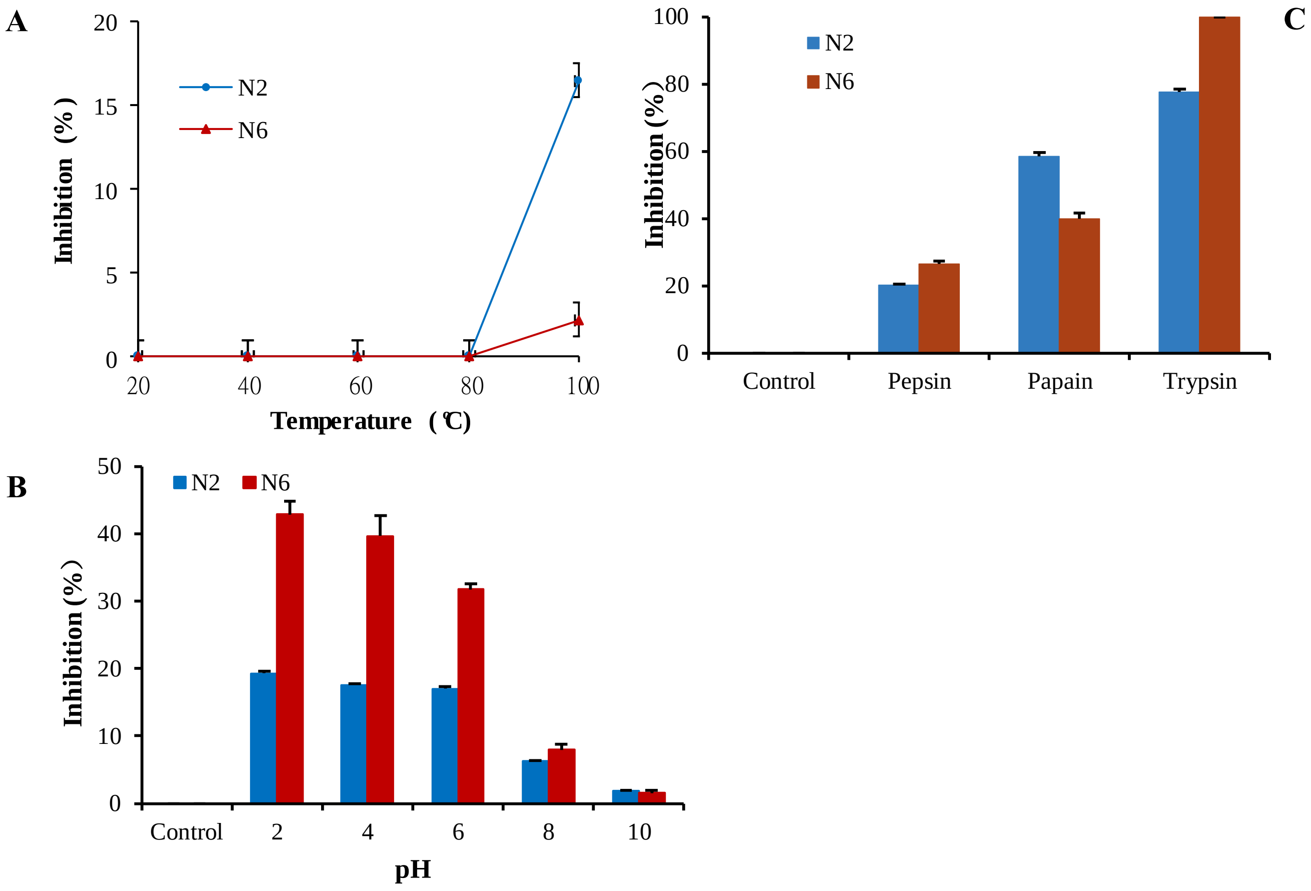

Figure 2 Stability of N2 and N6 against *E. coli* CVCC195. The effect of temperature **(A)**, pH **(B)**, and protease **(C)** on the antibacterial activity of N2, N6.

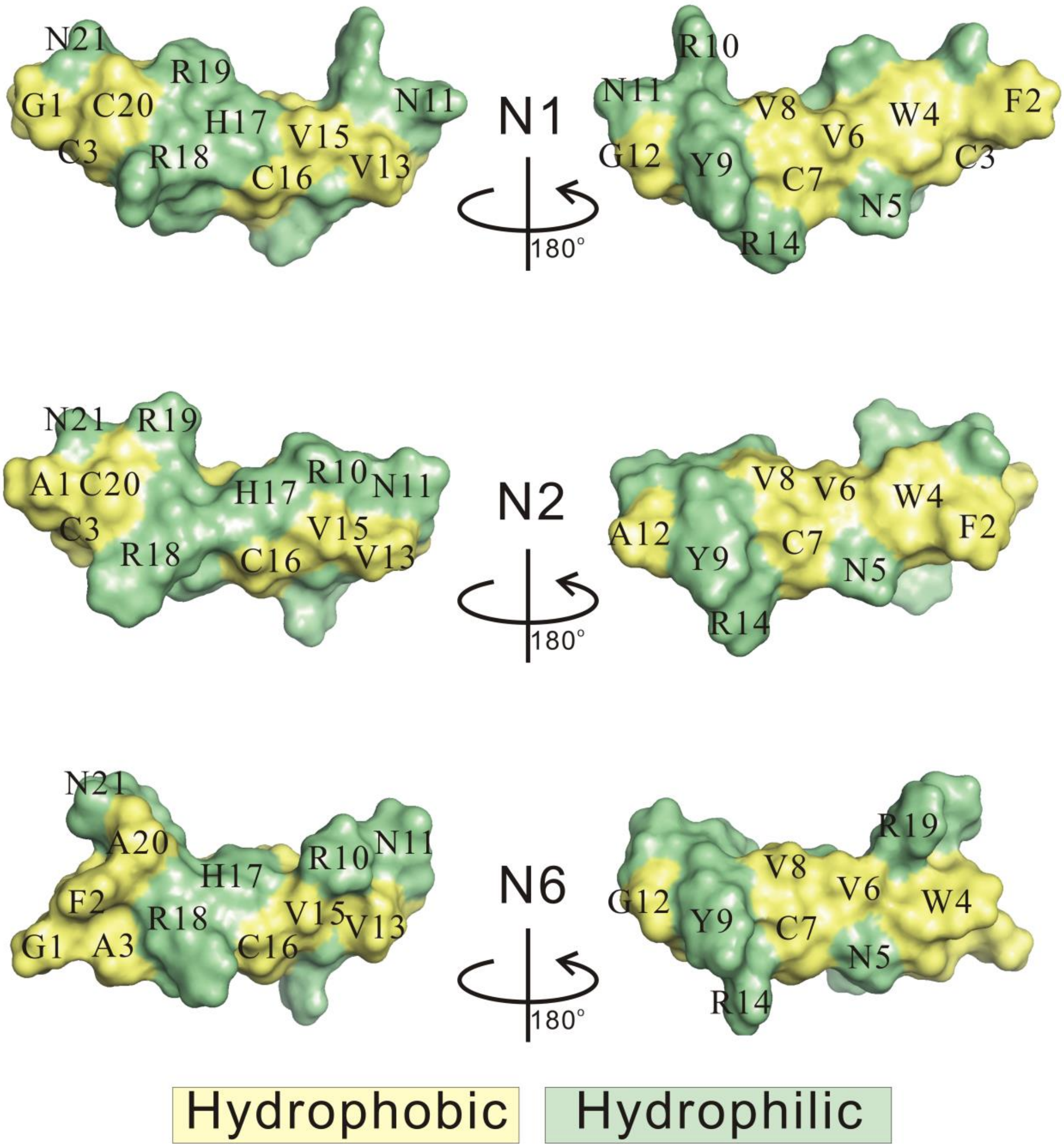

Figure 3 Two-side view of the three peptides structures with residue hydrophobicity on the surface. The yellow and green areas indicate hydrophobic and hydrophilic residues. The figure was prepared using program Pymol.

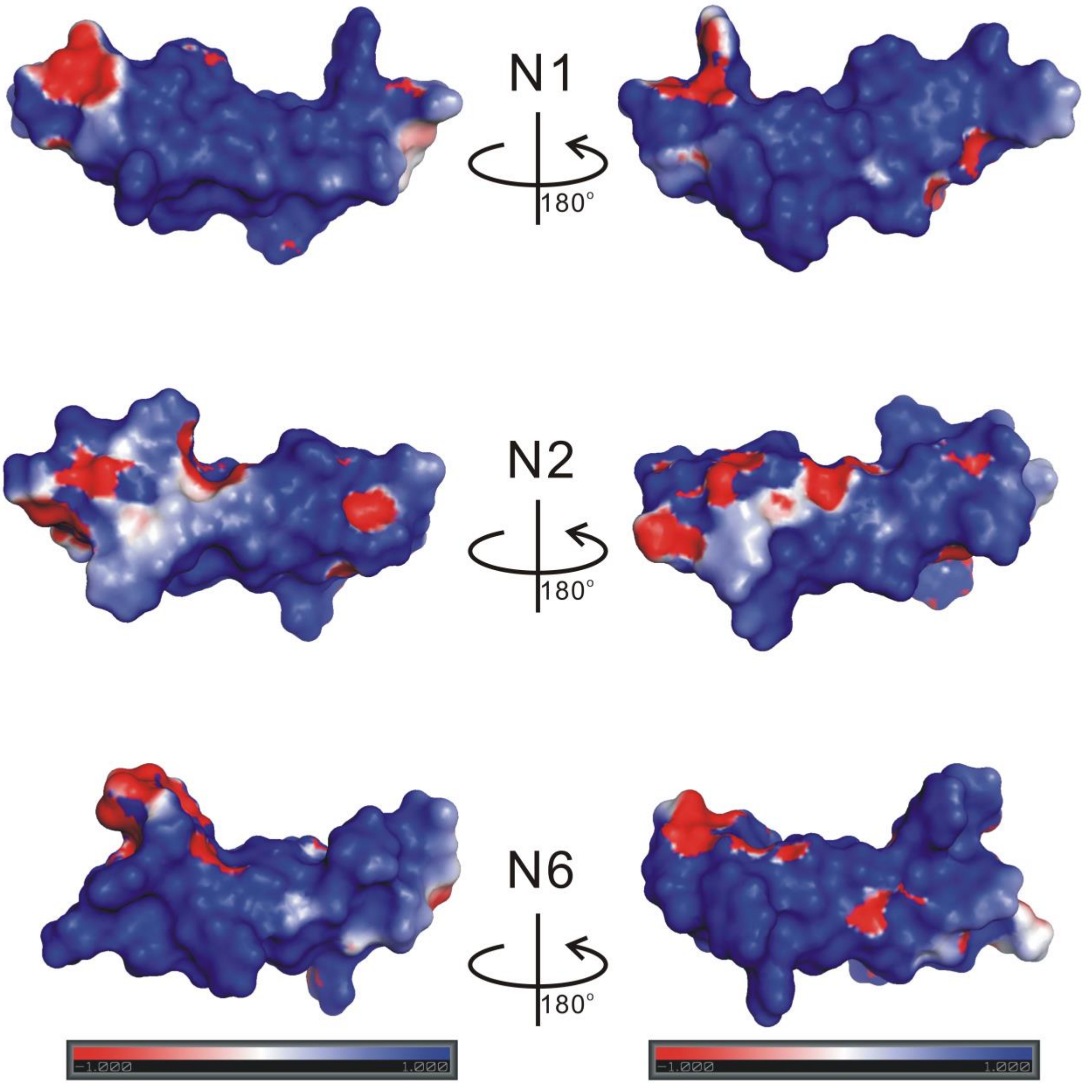

Figure 4 Two-side view of the three peptides structures with electrostatic potential on the surface. The blue and red areas denote positive and negative regions, respectively. The figure was prepared using program Pymol.

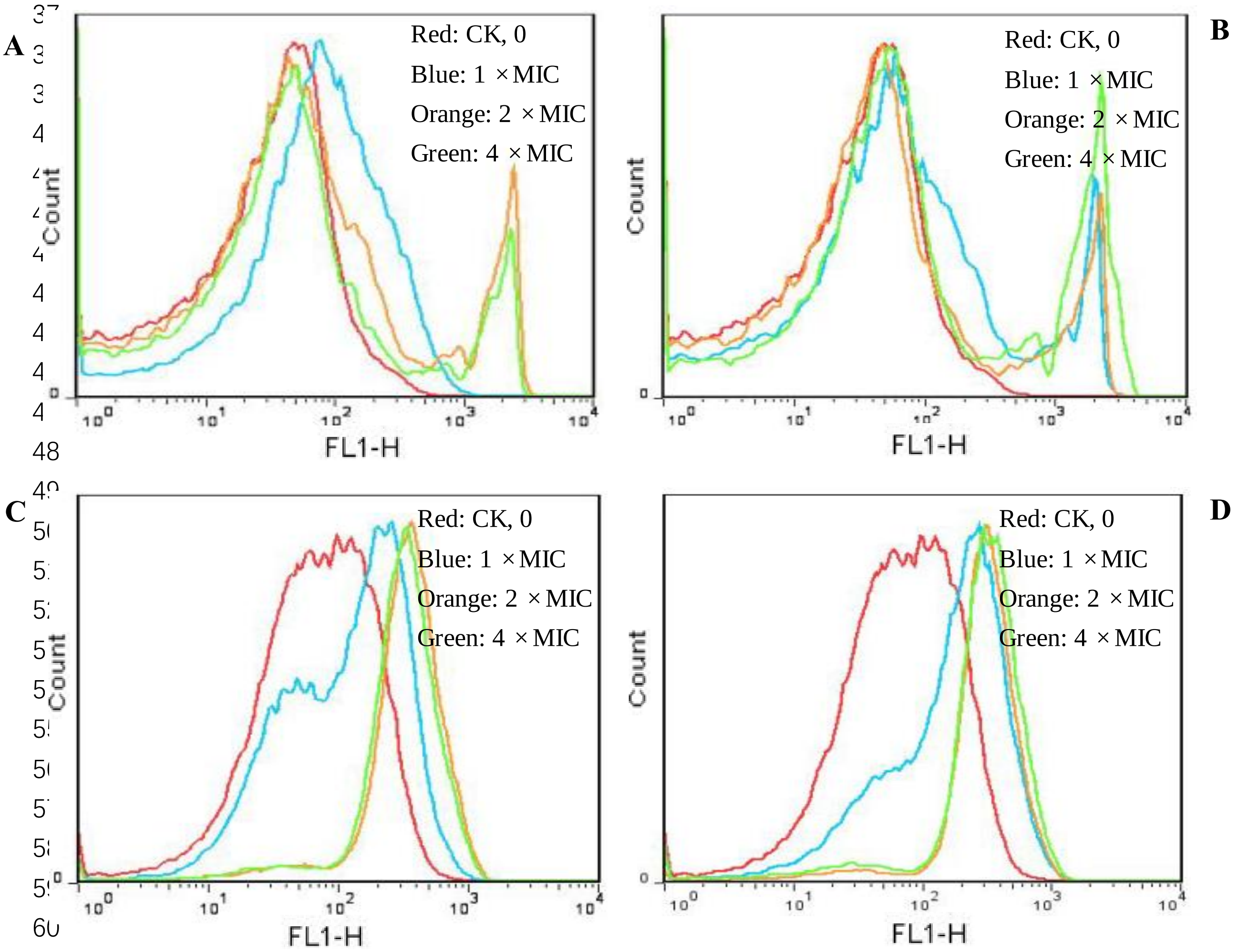

Figure 5 Inner membrane depolarization in *E. coli* and *S. enteritidis* treated with N2 and N6. Flow cytometric analysis of membrane potential of *E. coli* CVCC195 **(A, B)** and *S. enteritidis* CVCC3377 **(C, D)** induced by N2 **(A, C)** and N6 **(B, D)**, respectively. Red, no peptide, negative control; Blue, 1× MIC; Orange, 2× MIC; Green, 4× MIC. Bacteria in mid-logarithmic growth were treated with peptides for 2 h, stained with rhodamine-123, and analyzed on a flow cytometer.

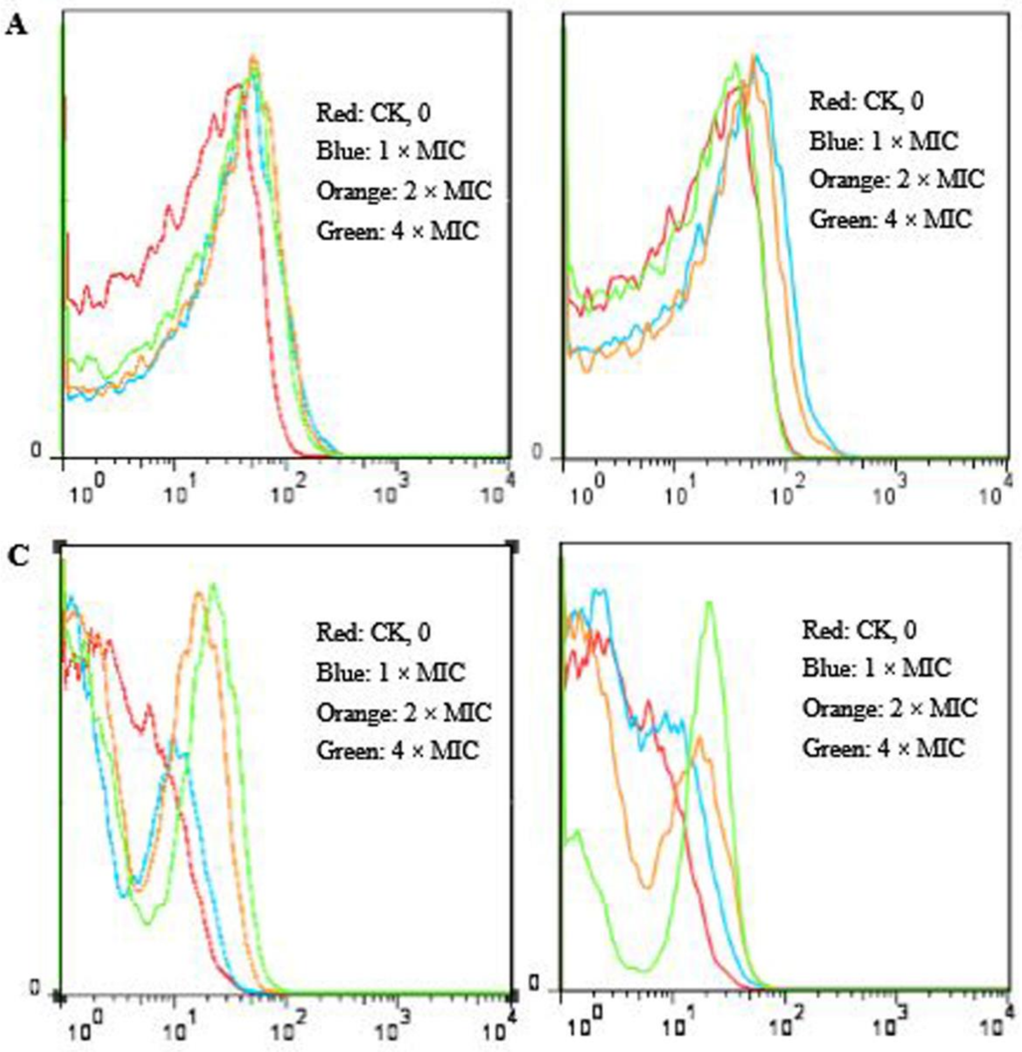

Figure 6 Intracellular ROS accumulation in the *E. coli* and *S. enteritidis* cells. **(A, B)** *E. coli* CVCC195. **(C, D)** *S. enteritidis* CVCC3377. Cells (10^8 CFU/ml) were cultured in the presence of 1 × MIC N2 **(A, C)** and N6 **(B, D)** for 2 h, respectively.

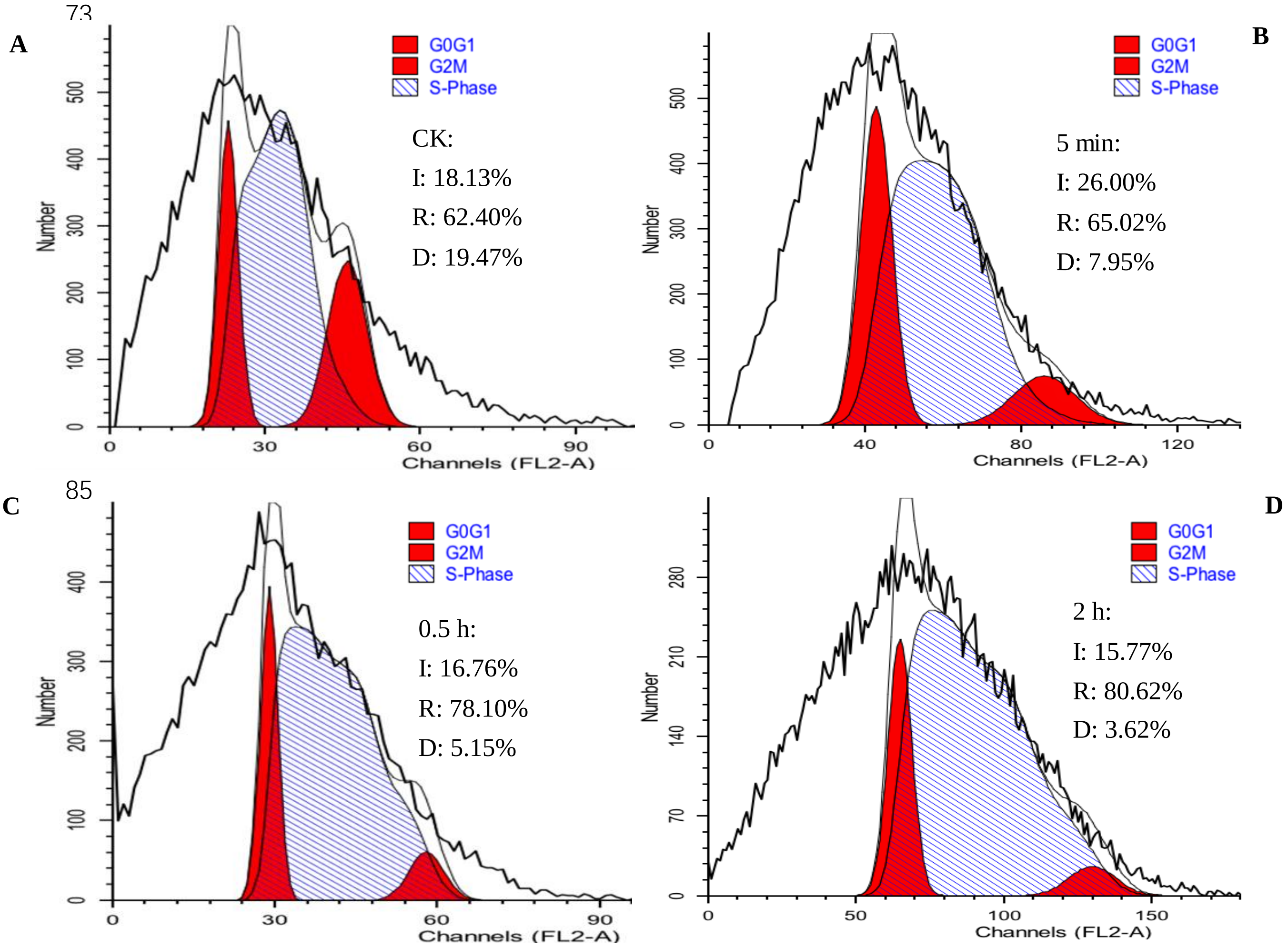

Figure 7 Effect of N2 on the cell cycle of *S. enteritidis* CVCC3377. Cells (10^8 CFU/ml) were cultured alone as a control group **(A)** or cultured in the presence of 1 × MIC N2 for 5 min **(B)**, 0.5 h **(C)**, and 2 h **(D)**, respectively.

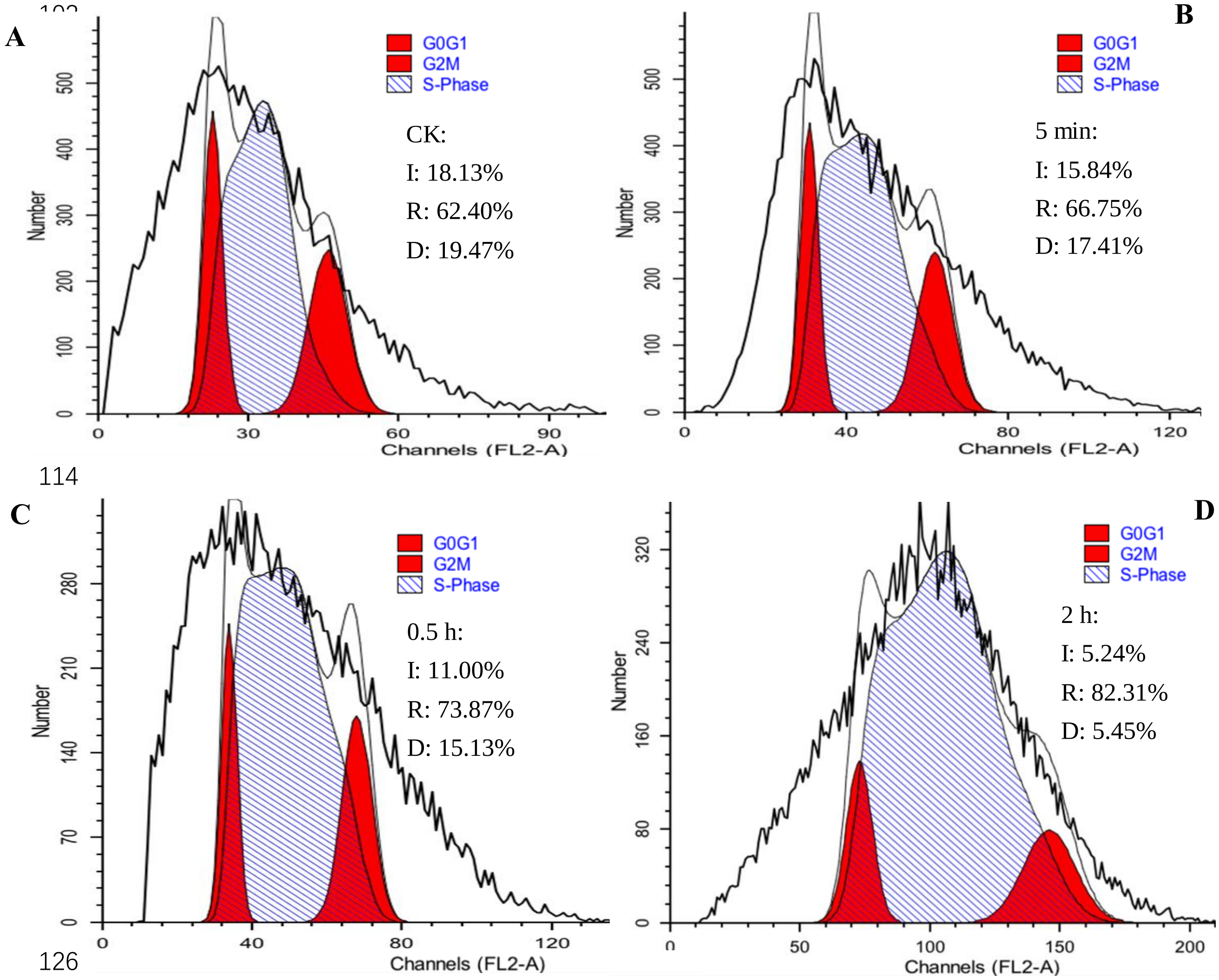

Figure 8 Effect of N6 on the cell cycle of *S. enteritidis* CVCC3377. Cells (10^8 CFU/ml) were cultured alone as a control group **(A)** or cultured in the presence of 1 × MIC N6 for 5 min **(B)**, 0.5 h **(C)**, and 2 h **(D)**, respectively.

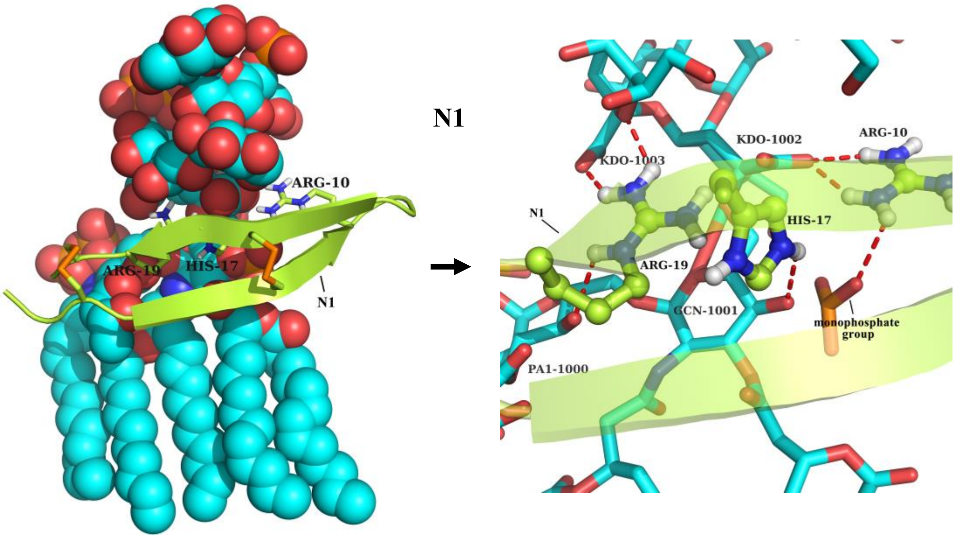

Figure 9 The complex structure of N1 and LPS (left) and detailed view of the binding pocket (right). N1 is shown as cartoon and the key residues (Arg10, His17, and Arg19) are shown as sticks and balls. The fatty acid chain GCN-1001 and the 2-Keto-3-deoxyoctonate ((KDO-1002 and KDO-1003) which belong to the core polysaccharide of LPS respectively form hydrogen bonds to His17 and Arg19, while Arg10 form a salt bridge with monophosphate group.

Supplementary 1: Table

Table 1 Analysis of 2D-structures of N2 and N6.

Secondary structure	The percentage of secondary structure in different solvents (%)											
	ddH_2O			10 mM SDS			20 mM SDS			40 mM SDS		
	N1	N2	N6	N1	N2	N6	N1	N2	N6	N1	N2	N6
Helix	12.3	22.0	19.6	54.6	89.3	85.2	51.4	92	81.1	46.7	80.9	80.3
Antiparallel	60.8	56.4	57.9	0.1	0	0	20.6	0	0	25.3	0	0.8
Parallel	2.0	0.4	0.5	5.4	1.0	4.9	2.8	0.8	2.0	3.3	2.0	4.1
β-turn	23.3	21.2	21.9	7.5	5.0	8.6	24.5	4.7	4.8	23.6	5.0	13.8
Random coli	1.6	0	0.1	32.4	4.7	1.3	0.7	2.5	12	1.1	12.1	1.0

Table 2 Temperature coefficient of amide protons.

Residue Number	Temperature Coefficient (ppb/K)		
	N1	N2	N6
1	N/A	N/A	N/A
2	-2.20	-3.86	-5.56
3	-5.59	-4.49	-6.92
4	-2.89	N/A	-5.84
5	-5.48	-5.38	-2.79
6	-3.83	-4.48	-7.42
7	-7.06	-6.99	-9.12
8	-1.08	-1.64	-2.43
9	-7.15	-7.25	-7.59
10	-3.70	-3.95	-4.71
11	-9.38	-10.00	N/A
12	-9.38	-7.48	-9.77
13	-2.40	-2.46	-2.36
14	-7.25	-9.05	-7.51
15	-4.74	-5.21	-6.00
16	-8.36	-8.54	-8.68
17	-1.41	N/A	-3.65
18	-5.23	N/A	N/A
19	-2.23	-2.40	-7.95
20	-2.67	-2.79	-7.94
21	-3.91	-5.24	-6.99

Table 3 Effect of N2 and N6 on the cell cycle of *E. coli* CVCC195.

	CK	N2			N6		
Phase		5 min	0.5 h	2 h	5 min	0.5 h	2 h
I-	33.4%	36.1%	41.9%	50.6%	33.8%	39.4%	46.5%
R-	41.3%	40.4%	38.8%	33.8%	41.3%	32.8%	30.2%
D-	22.0%	21.0%	9.4%	11.8%	17.7%	22.3%	13.0%
